# Molecularly cleavable bioinks facilitate high-performance digital light processing-based bioprinting of functional volumetric soft tissues

**DOI:** 10.1038/s41467-022-31002-2

**Published:** 2022-06-09

**Authors:** Mian Wang, Wanlu Li, Jin Hao, Arthur Gonzales, Zhibo Zhao, Regina Sanchez Flores, Xiao Kuang, Xuan Mu, Terry Ching, Guosheng Tang, Zeyu Luo, Carlos Ezio Garciamendez-Mijares, Jugal Kishore Sahoo, Michael F. Wells, Gengle Niu, Prajwal Agrawal, Alfredo Quiñones-Hinojosa, Kevin Eggan, Yu Shrike Zhang

**Affiliations:** 1 Division of Engineering in Medicine, Department of Medicine, Brigham and Women’s Hospital, Harvard Medical School, Cambridge, MA 02139 USA; 2 Department of Stem Cell and Regenerative Biology, Harvard University, Cambridge, MA USA; 3 Harvard Stem Cell Institute, Harvard University, Cambridge, MA USA; 4 Stanley Center for Psychiatric Research, Broad Institute of MIT and Harvard, Cambridge, MA USA; 5 University of the Philippines Diliman, Quezon City, Metro Manila Philippines; 6 Pillar of Engineering Product Development, Singapore University of Technology and Design, Singapore, Singapore; 7 Digital Manufacturing and Design Centre, Singapore University of Technology and Design, Singapore, Singapore; 8 Department of Biomedical Engineering, National University of Singapore, Singapore, Singapore; 9 Department of Biomedical Engineering, Tufts University, Medford, MA USA; 10 Departments of Neurosurgery, Oncology, Neuroscience, Mayo Clinic, Jacksonville, FL USA

**Keywords:** Materials science, Biomedical engineering, Tissue engineering, Biomaterials

## Abstract

Digital light processing bioprinting favors biofabrication of tissues with improved structural complexity. However, soft-tissue fabrication with this method remains a challenge to balance the physical performances of the bioinks for high-fidelity bioprinting and suitable microenvironments for the encapsulated cells to thrive. Here, we propose a molecular cleavage approach, where hyaluronic acid methacrylate (HAMA) is mixed with gelatin methacryloyl to achieve high-performance bioprinting, followed by selectively enzymatic digestion of HAMA, resulting in tissue-matching mechanical properties without losing the structural complexity and fidelity. Our method allows cellular morphological and functional improvements across multiple bioprinted tissue types featuring a wide range of mechanical stiffness, from the muscles to the brain, the softest organ of the human body. This platform endows us to biofabricate mechanically precisely tunable constructs to meet the biological function requirements of target tissues, potentially paving the way for broad applications in tissue and tissue model engineering.

## Abstract (web summary)

Soft tissue fabrication using digital light processing remains challenging. Here the authors present a molecular cleavage approach to achieve high-performance bioprinting of constructs with tissue-matching mechanical properties and structural complexity.

## Introduction

Additive biomanufacturing, commonly known as three-dimensional (3D) bioprinting, has drawn on continuously increasing attention in tissue biofabrication for a range of applications^1–5^. In particular, proper integration of 3D bioprinting with meticulously designed bioinks is necessary to meet multifactorial mechanical and physiological requirements optimized for tissue generation. To date, significant progress has been made towards producing structurally sophisticated constructs featuring tissue-mimicking shapes and geometries using various 3D bioprinting modalities^6,7^. Notably, 3D bioprinting using the digital light processing (DLP)-based approach oftentimes exhibits a superior performance in terms of both printing speed as well as structural complexity compared to other bioprinting methods, such as extrusion-based techniques^8–10^. For example, bioprinting of hydrogel models of a distal lung-mimicking construct and vessel-like microchannel-embedded constructs, among others, were recently reported through the use of DLP-based (bio)printing^11^.

Although structural complexity plays a vital role in tissue recapitulation, physiological microenvironments are also essential to be considered to attain proper tissue-specific functions^12^. In fact, very little optimization work has been carried out to formulate enabling bioinks for DLP bioprinting, which should meet the mechanical demand of printing fidelity while simultaneously satisfying the biological requirements for the loaded cell types^13^. In particular, it remains highly challenging to construct soft organ-mimics such as the brain and the liver^14^. As a matter of fact, the prerequisites in bioink development for soft-tissue biofabrication are normally mutually exclusive across the different bioprinting modalities. On the one hand, bioinks with strong mechanical properties can aid in filament deposition in extrusion bioprinting or layer-by-layer lifting in DLP bioprinting^15^. Nevertheless, the stiff bioink networks result in limited cellular functions, including but not limited to cell spreading, proliferation, and differentiation, for cells that are of soft tissues in origin^16,17^. On the other hand, the mechanical properties of bioinks that are compatible with soft tissue-derived cells are usually insufficient to facilitate the bioprinting process, especially when volumetric constructs are desired^9,18^. This dilemma is well-exemplified by the vast body of currently existing reports on DLP bioprinting; high-fidelity, volumetrically sophisticated structures with limited bioactivities are obtainable when the (bio)ink mechanics are high^11^, whereas soft bioinks with good cellular activities would only allow creation of planar or pseudo-3D tissue constructs^19,20^. Therefore, the lack of cytocompatible yet mechanically tunable bioink design remains as a major inhibitor for further applications of DLP bioprinting of truly 3D, structurally and biologically relevant tissue constructs.

To overcome this obstacle, some strategies have been proposed through modulating different photocrosslinking ratios in DLP-based bioprinting, where soft constructs with mechanical properties as low as 10 kPa were obtained^21^. Further exploration has been made via the utilization of a fluid support, where a support fluid immiscible with bioinks were used to provide extra buoyant forces for printing^22^. Nevertheless, the lowest mechanical properties of bioprinted constructs with this method were still limited to ~7 kPa, in addition to the concerns associated with the extremely complicated instrumentation setup. Furthermore, uncertainty remains as to the instable bioprinting process due to the presence of the liquid interface, as well as the unwanted contamination by the immiscible support fluid. These strategies have achieved a few of the above requirements, such as volumetric printability and some degrees of cytocompatibility. Other progress has been made with a bioink of complementary thermoreversible gelatin network in extrusion-based bioprinting, whereby the bioprinted constructs could meet biological requirements of loaded cells via the sacrifice of the gelatin molecules^18^. Despite many efforts been exerted so far, the reconstitution of ultrasoft tissues through DLP bioprinting still needs notable progress.

**Fig. 1:**
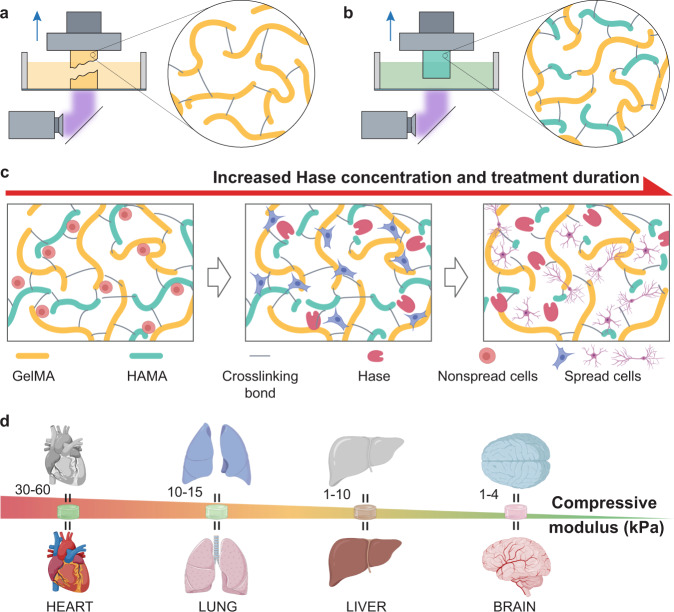
High-fidelity DLP bioprinting using the GelMA/HAMA bioinks featuring both enhanced printability and widely tunable mechanical properties. **a** Schematic of the DLP-based 3D bioprinting with conventional, pure GelMA bioinks, where bioprinting would fail because of the insufficient mechanical properties when the bioinks are used at low concentrations. **b** Schematic of the DLP-based 3D bioprinting showing high-fidelity fabrication using the GelMA bioinks homogeneously mixed with HAMA. **c** Enzymatic digestion procedure of the GelMA/HAMA-bioprinted constructs with elevated Hase concentration and treatment duration, enabling increased cleavage of HA, reducing matrix stiffness, and promoting cell spreading and functions. **d** Tissue-mimics bioprinted with the GelMA/HAMA bioinks could be obtained by precisely controlling the post-bioprinting digestion of the HAMA component to achieve target tissue-matching mechanical properties across a wide range.

In this study, we attempt to address this unmet need of bioink development through a sufficiently simple yet highly efficient materials approach, via the use of target bioinks (*e.g*., gelatin methacryloyl (GelMA) as a demonstration) homogeneously mixed with hyaluronic acid (HA) methacrylate (HAMA), both photoactive, to enable DLP bioprinting with simultaneous structural fidelity and bioactivity (Fig. 1). This method generates good volumetric printability due to the high mechanical performance of the HAMA-mixed GelMA bioink during bioprinting. Of note, post-enzymatic digestion of the HAMA molecules using hyaluronidase (Hase) subsequently leads to final constructs with well-maintained structural fidelity. More importantly, desired mechanical properties within a wide range (above 100 kPa down to around 1 kPa), are precisely tunable by the enzymatic digestion process. A mechanical property library in relation to bioink formulations and digestion parameters is further established through mathematical modeling. We demonstrate the wide applicability of this approach by bioprinted multiple functional soft tissues from the muscles to of utmost excitement, the brain, the softest organ of the human body^23^, all in truly volumetric, structurally sophisticated architectures. This design strategy of mechanically tunable, molecularly cleavable bioinks presents an enabling means to customize the mechanical and biological properties of DLP-bioprinted tissue constructs towards creating not only biomimetic structures but also tissue-matching mechanical microenvironments for ultimate functionalities.

## Results and discussion

### Printability of GelMA bioinks mixed by HAMA

GelMA, as a photocrosslinkable derivative of gelatin, possesses advantages of favorable cyto/biocompatibility, intrinsic bioactive motifs, and controllable on-demand gelation kinetics^16,24–26^. GelMA-based bioinks, including hybrid bioinks of GelMA and HA-derivatives, have been widely used for extrusion-based bioprinting and tissue engineering applications, including those that are soft in nature thanks to the recently popularized embedded bioprinting strategy^27–30^. However, it is still challenging to obtain soft 3D hydrogel constructs of GelMA (or any other similar bioinks) via the DLP-based bioprinting technique, due to its weak mechanical properties at low concentrations, which are insufficient to support the gravity of layer-by-layer 3D-fabricated structures in particular in the bottom-up configuration (Supplementary Fig. 1). For example, the 10% (all expressions are in w/v% unless otherwise noted) GelMA was capable of being DLP-printed into a 3D cubic construct. In comparison, the reduced concentration of GelMA at 7.5% resulted in only partial printing, as evidenced by the break in the middle of the cube (Supplementary Fig. 2). As expected, the printing failed from the very first layer when the GelMA concentration was further decreased to 5.0%.

While it is possible, in principle, to produce relatively soft hydrogel constructs with the top-down DLP setup, it also does not support the (bio)printing of high-fidelity patterns due to the surface tension effect, in addition to the significant waste of bioink that has to fill the entire vat during the fabrication process^13^. Computed axial lithography (CAL) is another recent technique that allows efficient decoupling of mechanical performances of bioinks and bioprinting shape fidelity^31,32^. However, its build volume can be limited due to the need for light penetration through the entire bioink tank (many bioinks/photoinitiators are absorption-bearing). In comparison, DLP-based (bio)printing enables fabrication of large-volume constructs due to its layer-by-layer photocrosslinking nature^33–35^.

We formulated a collection of GelMA/HAMA inks with different concentrations as well as molecular weights (*M*_w_) of HAMA (10–1500 kDa), and explored their volumetric printability using DLP. The HAMA and GelMA used in this study were analyzed by ^1^H nuclear magnetic resonance spectra (NMR, Supplementary Fig. 3 and Supplementary Fig. 4), to confirm successful modification. Specifically, the degree of methacryloyl-substitution for GelMA was determined at 81.7 ± 0.7% (Supplementary Fig. 4). In addition, the primary amine contents were measured by the trinitrobenzene sulfonic acid (TNBSA) assay, which for gelatin and GelMA were 8.1 ± 0.2 µmol mg^−1^ and 1.5 ± 0.1 µmol mg^−1^, respectively.

**Fig. 2:**
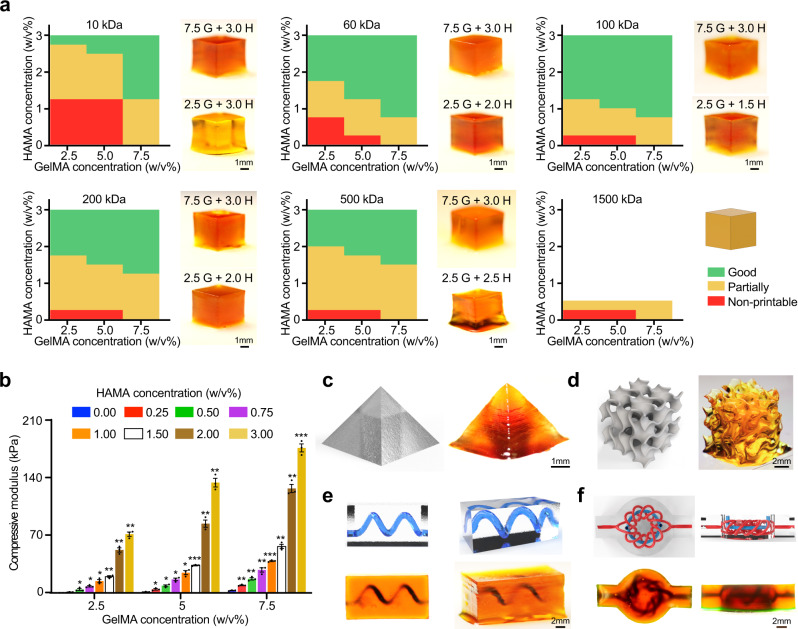
Printability assessments of the GelMA/HAMA inks in DLP-based 3D printing. **a** Printability maps and representative images of 3D-printed cubes using diverse enzyme-digestible inks, covering 2.5%, 5.0%, or 7.5% GelMA, and 0 to 3% HAMA (*M*_w_ = 10, 60, 100, 200, 500, or 1500 kDa). **b** Compressive moduli of samples fabricated from GelMA/HAMA inks across various formulations. *n* = 3; one-way ANOVA; *p < 0.05, **p < 0.01, ***p < 0.001 (compared with the respective control groups of 0% HAMA). Data are presented as mean values ± SDs. **c**–**f** Photographs showing 3D-printed constructs featuring sophisticated shapes and internal structures, using the GelMA/HAMA ink of 5.0%/3.0% (*M*_w_ of HAMA = 100 kDa) and corresponding digital models used for printing. **c**, Pyramid in the side view. **d** Gyroid in the side view. **e** Cuboid containing a spiral, perfusable channel in the top view and side view. **f** Torus knot (the digital design was obtained from^11^) in the top view and side view. **a**, and **c**–**f**, images are representatives of *n* = 3 independent experiments. G GelMA, H HAMA. Source data are provided as a Source Data file.

A cube with a size of 4 × 4 × 4 mm^3^ was printed by each formulation of the inks with a 100-μm layer thickness and a 30-s crosslinking time for each layer. The printability maps that we generated accordingly indicated that the printable areas (*i.e*., number of green squares in each map) were in general enlarged when HAMA concentrations were increased, at the *M*_w_ under 500 kDa (Fig. 2a). Among these, printability of the inks containing HAMA of *M*_w_ = 10 kDa and *M*_w_ = 500 kDa exhibited limited areas on the maps because of their weak mechanical properties and high viscosity values, respectively. Moreover, the inks containing HAMA (*M*_w_ = 1500 kDa) displayed completely non-printable features due to their overly high viscosities. On the contrary, the largest green printable areas in this set of printability maps represented HAMA of *M*_w_ = 100 kDa, endowing better printability compared with the inks containing 60-kDa or 200-kDa HAMA. A similar trend of printability variations was observed when we attempted to increase or decrease the GelMA concentrations in the inks as well. Therefore, an optimal HAMA *M*_w_ of 100 kDa was selected for the subsequent experiments, allowing successful 3D printing of the inks covering 2.5% GelMA homogeneously mixed with HAMA from 1.25 to 3.00%, 5.0% GelMA with HAMA from 1.00 to 3.00%, and 7.5% GelMA with HAMA from 0.75 to 3.00%. As noted above, GelMA inks at these concentrations (2.5–7.5%) by themselves, are not directly printable with the DLP method (Supplementary Fig. 2) unless mixed with other photocrosslinkable components (*e.g*., poly(ethylene glycol)-diacrylate (PEGDA)^11^ or methacrylated poly(vinyl alcohol) (PVA-MA)^36^) again resulting in stiffer hydrogels. Though, even printable in the presence of these additives, the activities of embedded cells would likely become strongly affected by the densely crosslinked polymer networks.

The compressive moduli of the constructs fabricated with GelMA/HAMA (HAMA *M*_w_ = 100 kDa) inks were further evaluated. As shown in Fig. 2b, the mechanical properties were augmented with the aid of inclusion of HAMA molecules. Taking 5.0% GelMA as an example, the compressive moduli improved from 1.3 ± 0.2 to 134.4 ± 3.1 kPa when the HAMA concentration was increased from 0.0 to 3.0%. Similar tendencies were observed for 2.5% GelMA and 7.5% GelMA. These results of mechanical properties were in accordance with what we obtained from the printability maps, where the formulated inks featuring compressive moduli >20 kPa generally exhibited good printability in the DLP-based method.

We subsequently investigated the printing parameters for the ink made of GelMA/HAMA (5.0%/3.0%), which was the representative ink formulation with good printability. We explored the influence of photoabsorber and exposure time on the curing depth of the ink (GelMA/HAMA, 5.0%/3.0%). In DLP (bio)printing, photoabsorber is oftentimes used to attenuate excessive light and adjust the photopolymerization kinetics to achieve the desired layer thickness, benefiting the printing fidelity^37^. Ponceau 4R was chosen as the photoabsorber in our visible light-based bioprinting system, since its absorbance spectrum is identified to encompass the visible-light wavelengths^38^. The successful control over the optical penetration length by addition of Ponceau 4R and its cytocompatibility have been proven in our previous studies^39–42^. The working curve represents the photopolymerization kinetic of the ink and serves as a quick estimation of printing settings. We plotted the working curves of the inks made of GelMA/HAMA (5.0%/3.0%) via Eq. (1):

1 $${C}_{d}={D}_{p}\,{ln}\frac{E}{{E}_{c}}$$

where *C*_*d*_ is the cure depth, *D*_*p*_ is the light penetration depth, *E* is the irradiation dosage, and *E*_*c*_ is the energy required for achieving the gelation point. The irradiation dosage was calculated from light intensity multiplied by exposure time, and the curing depth directly measured from the optical microscopy image of the printed construct (Supplementary Fig. 5a). As shown in Supplementary Fig. 5b, c, for the ink of GelMA/HAMA (5.0%/3.0%) without photoabsorber, the curing depths ranged from 576.0 ± 32.7 to 1162.3 ± 67.0 µm when the crosslinking times were changed from 5 to 30 s. By adding the photoabsorber, the curing depths could be gradually adjusted to 104.7 ± 12.3 to 633.3 ± 24.3 µm (1% Ponceau 4R), 34.7 ± 3.7 to 354.0 ± 16.1 µm (2% Ponceau 4R), and 21.7 ± 3.9 to 184.0 ± 11.3 µm (3% Ponceau 4R). Considering the thickness demands of our printing were 50 to 300 µm, we chose 2% Ponceau 4R for further investigations.

Moreover, a radial pattern was utilized to assess the resolution at different crosslinking times with or without photoabsorber (2.0% Ponceau 4R) addition (Supplementary Fig. 6). The diameters of the central circles were raised when the exposure time was extended from 5 to 30 s, likely due to the fast diffusion of the free radicals, which was conversely reduced in the presence of photoabsorber to the inks. Meanwhile, the calculated resolution, which implied the smallest distance that could be distinguished between two independent points, was enhanced after incorporating the photoabsorber. The influence of exposure time on the resolution of the printed patterns was not as significant in the inks containing the photoabsorber compared to those without. Among all the parameters we evaluated, the radial diameter of 1.1 mm and the resolution of 200 µm, which were closest to our designed pattern, could be obtained using 15 s of exposure time and 2.0% of photoabsorber concentration.

By utilizing the optimized ink formulation and printing conditions (100-µm thickness and 15-s exposure time for each layer), we printed several representative 3D constructs featuring complex internal and external architectures. It was observed that a solid pyramid with clear sharp edges could be printed (Fig. 2c). Additionally, a gyroid featuring highly curved surfaces and interconnected internal pores was printed to demonstrate the ability to generate 3D porous scaffolds (Fig. 2d). We also confirmed the capacity to print 3D constructs having complex embedded channels, including the structure with a spiral channel (diameter = 1 mm, Fig. 2e) and the structure containing a torus channel entangled with another torus knot-like channel (Fig. 2f)^11^. Perfusions with a color dye verified the pattern fidelities and the channel patency. These 3D geometries encompassing challenging features (sharp edges, interconnected pores, and sophisticated hollow channels) are highly desirable in tissue biofabrication applications, exhibiting again the printability of our GelMA inks homogeneously mixed by HAMA.

### Enzymic digestion of HAMA and cytocompatibility assays

Hase can catalyze the hydrolysis of the *β*-1, 4-glycosidic bond between hyaluronic acid monomers^43^. The concentration and duration of Hase treatments were anticipated to play primary roles in the precisely tunable mechanical properties of the DLP-printed constructs. As shown in Supplementary Figs. 7–8, the mechanical properties of GelMA/HAMA constructs decreased when the Hase concentration or digestion time was increased. This phenomenon was most clearly identified with GelMA/HAMA at 7.5%/1.5%, which endorsed a relatively high initial stiffness (56.6 ± 2.3 kPa) that was significantly reduced to 10.1 ± 0.6 kPa after enzymatic digestion to a value slightly >7.5% pure GelMA (3.0 ± 0.5 kPa). It should be pointed out once more that the 7.5% GelMA by itself is almost non-printable through the DLP method (Supplementary Fig. 2). More importantly, a wide range of compressive moduli could be achieved through the digestible HAMA network, from ~180 kPa all the way down to 1 kPa, which is suitable for modeling multiple soft tissues including but not limited to the brain (1–4 kPa), the liver (1–10 kPa), the lung (10–15 kPa), and the heart (30–60 kPa)^44^.

Fluorescein isothiocyanate (FITC)-conjugated HAMA-mixed GelMA was used to further confirm the enzymatic cleavage of HAMA. As shown in Supplementary Fig. 9, microscopic images presented clear digestion signs under different Hase concentrations after 24 h of incubation. The decreased fluorescence intensity suggested that a rapid digestion occurred under a higher Hase concentration and a lower concentration of GelMA. For instance, the disc printed with 2.5% GelMA mixed with 1.5% HAMA displayed a near-complete digestion after 24 h of Hase treatment at the concentration of 1000 U mL^−1^. Another digestion analysis used consistent Hase concentration (1000 U mL^−1^) but varied treatment durations (Supplementary Fig. 10). The results indicated that the digestion time also played a key role in HAMA cleavage, which coincided with the mechanical test results as well. In the process of Hase digestion, the cleavage of HAMA generated smaller pieces of disaccharide chains that were leached out. This was the possible reason that some of the labeled-HAMA was observed to diffuse out with digestion. Therefore, the crosslinked HAMA content was reduced, and the overall crosslinking density was decreased, leading to the lowered and tunable mechanical stiffness of the printed constructs following Hase treatment.

Further investigations regarding Hase diffusion within the GelMA/HAMA (2.5%/1.5%) hydrogels (Supplementary Fig. 11a) exhibited that FITC-dextran (*M*_w_ = 60 kDa), the replacement of Hase (*M*_w_ = 55 kDa) for easier visualization, had an effective diffusion coefficient of 3.5 × 10^−8^ cm^2^ s^−1^, as calculated by tracking the axial diffusion. In reality, however, with the Hase digestion undergoing, its diffusion rate in the GelMA/HAMA hydrogel would be dynamic since the digested hydrogel network could also influence the overall diffusion. Indeed, when we conducted the same diffusion test using the Hase-treated (1000 U mL^−1^ for 24 h) GelMA/HAMA samples as an example, we did observe a faster FITC-dextran diffusion speed compared with the untreated control (Supplementary Fig. 11a). The effective diffusion coefficient was also calculated to elevate to 1.8 × 10^−7^ cm^2^ s^−1^ in the Hase-digested GelMA/HAMA hydrogels (Supplementary Fig. 11b). To this end, once knowing such diffusional parameters in different relevant setups, it would be convenient to precisely design the conditions that would allow to achieve uniform diffusion of Hase and digestion.

To fully understand the influence of ink formulation and Hase administration, a mathematical model was established to predict the compressive moduli of the digested constructs based on experimental results, using Eq. (2) expressed below:

2 $${Compressive}\,{modulus}\left({kPa}\right)=\,	-113.3016+5.8599{GelMA}\\ 	-0.1708{Digestion}\,{time}-0.0173{Hase}\\ 	+66.1107{HAMA}-0.3595{GelMA}\times {Digestion}\,{time}\\ 	-0.0099{GelMA}\times {Hase}+6.9941{GelMA}\times {HAMA}\\ 	-2.1007{Digestion}\,{time}\times {HAMA}\\ 	-0.0458{Hase}\times {HAMA}+0.1663\,{{Digestion}{time}}^{2}\\ 	+0.0001{{Hase}}^{2}$$

The equation illustrates that the HAMA concentration was the most significant factor in determining the moduli with a coefficient of 66.11 kPa × %HAMA^−1^. This was followed by the product of GelMA and HAMA (6.9941% GelMA^−1^ × %HAMA^−1^) and then by the GelMA concentration (5.8599% GelMA^−1^). However, it should be noted that, this equation should not be used to determine the relative impact of each factor because the coefficients were scaled to accommodate the units of each factor and the intercept was not at the center of the designed space. To determine the relative impact of each term, the model equation in terms of coded values is given below in Eq. (3), which was able to make predictions regarding the responses for given coded levels of each factor.

3 $${Compressive}\,{modulus}\left({kPa}\right)=\;	28.76+30.87A-32.44B-31.7C+39.73C\\ 	-10.79A\times B-12.33A\times C+13.11A\times D-18.91B\times D\\ 	-17.18C\times D+23.95\,{B}^{2}+26.58\,{C}^{2}$$

where *A* is the concentration of GelMA (%), *B* is the digestion time of Hase (h), *C* is the concentration of Hase (U mL^−1^), and *D* is the concentration of HAMA (%). By default, the high levels of the factors were coded as +1 and the low levels were coded as −1. The actual values were then scaled using this range. The coded equation was useful for identifying the relative impacts of the factors by comparing the factor coefficients. The statistical significances of the fitted second-degree polynomial model were assessed by the *P*-value of analysis of variance (ANOVA), which are given in Supplementary Table 1.

**Fig. 3:**
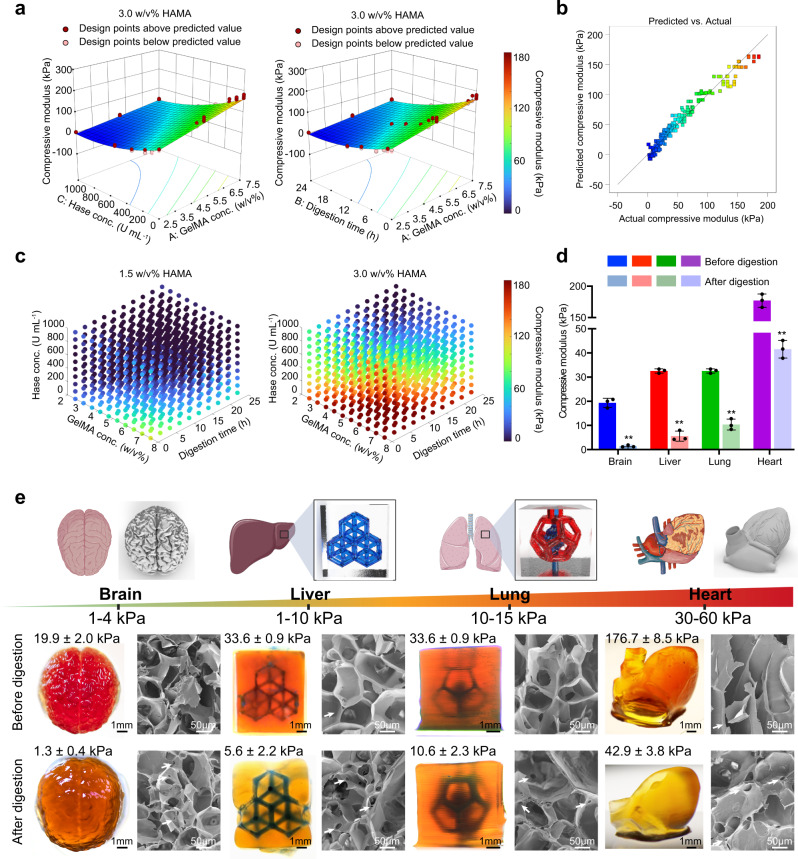
Widely tunable mechanical properties of the constructs bioprinted from GelMA/HAMA bioinks and their feasibility to fabricate structurally sophisticated soft tissue-mimics. **a** 3D surface plots showing the effects of different parameters on mechanical properties of the final constructs made from 3.0% HAMA in combination with different concentrations of GelMA and Hase. **b** Comparison plot between predicted and experimental moduli of Hase-digested constructs. **c** Parameter maps showing the relationships between compressive moduli and bioink formulations, including GelMA concentration, Hase concentration, and the digestion time. **d** Compressive moduli of constructs printed with different ink formulations to achieve tissue-matching mechanics post-enzymatic digestion, where the digestion parameters were determined from the mathematical model. *n* = 3; one-way ANOVA; **p < 0.01 (compared with the respective groups of before digestion). Data are presented as mean values ± SDs. **e** 3D tissue-mimics printed with 2.5%, 5.0%, 5.0%, or 7.5% GelMA containing 1.5% or 3.0% HAMA to emulate the brain, liver, lung, or heart, respectively. Bottom rows show photographs of the printed structurally sophisticated constructs before and after different parameters of Hase digestion and the corresponding SEM images. Images are representatives of *n* = 3 independent experiments. Source data are provided as a Source Data file.

From the results, the coded HAMA concentration term displayed the highest impact on controlling the modulus value followed by digestion time, Hase concentration, and GelMA concentration. Surface plots of the model were determined by setting the Hase concentration to 1000 U mL^−1^ and the HAMA concentrations to 1.5% (Supplementary Fig. 12) or 3% (Fig. 3a). Analyses of the fit statistics revealed that the model had a coefficient of determination R^2^ equal to 0.9590 and an adequate precision value of 78.28, suggesting an excellent fit and thus could be used to navigate the design space. Figure 3b revealed the graph of the predicted values *versus* experimental values, whereby the closer a particular point was to the diagonal line, the better of the prediction. This plot indicated that prediction using this model was better at relatively low values of modulus (<130 kPa) than at higher modulus values (>130 kPa). Figure 3c elucidated the establishment of parameter maps, by which any targeted moduli of mimic tissues could be navigated to find the initial concentrations of GelMA/HAMA to print, as well as the conditions of Hase concentration and digestion times for post-printing treatment, suggesting a good potential for multiple soft-tissue designs and fabrications. We further conducted additional verification experimental trials according to the predicted outcomes based on this established mathematical model. Target modulus values of 5, 15, 30, 65, and 110 kPa were chosen and the parameters used were calculated using numerical optimizations. This broad modulus range roughly covered our desired tissue moduli. The prediction interval was calculated beforehand to estimate an interval in which the mean of the additional trials would fall, at a probability of 95%. As shown in Supplementary Table 2, all five experimental moduli were found within the range of the respective prediction intervals (PIs), verifying the success of this simulation model.

Considering that this mathematical modeling approach provided distinct information of bioink formulation and digestion conditions of any target mechanical property, we selected three bioink compositions from this simulation to fabricate desired tissue-mimics, including the brain (GelMA/HAMA, 2.5%/1.5%), the liver or the lung (GelMA/HAMA, 5%/1.5%), and the heart (GelMA/HAMA, 7.5%/3.0%). As shown in Fig. 3d, the mechanical properties of the typical soft tissues in the human body, such as the brain, liver, lung, and heart, were recapitulated through different bioink formulations and their digestion parameters determined from the mathematical model. In these examples, the compressive moduli of the brain-mimic changed from 19.9 ± 2.0 kPa (immediately post-fabrication) to 1.3 ± 0.4 kPa (post-digestion of HAMA), liver-mimic from 33.6 ± 0.9 kPa to 5.6 ± 2.2 kPa, lung-mimic from 33.6 ± 0.9 kPa to 10.6 ± 2.3 kPa, and heart-mimic from 176.7 ± 8.5 kPa to 42.9 ± 3.8 kPa. All the mechanical properties of printed tissue-mimics were widely tuned following this procedure and achieved proper range of target stiffness. On the basis of these results, we concluded that this strategy could be used to precisely tune the mechanical properties of DLP-printed constructs through enzymatic digestion within acceptable prediction intervals.

In addition, we successfully mimicked various exemplary complex shapes (brain and heart) as well as the representative vascular patterns (liver and lung) with these constructs using DLP printing (Fig. 3e). The printed brain-like structure displayed clearly visible cerebral sulci and grooves. The heart-like construct featured the 3D anatomical shape containing the aorta and the left and right ventricles. Excellent printability and perfusable performances were also observed in the designs of the liver and the lung, whereby the truly volumetric hexagonal lobule units (also see Supplementary Fig. 13) and the bioinspired alveolar structure each contained an embedded 3D anatomical vascular network. Of particular note, all the printed fine features were retained after enzymatic digestions, where the diameter of the embedded microchannels such as those in the hepatic lobule, the alveolar lung, and the cuboid became slightly enlarged post-digestion (Fig. 3e, Supplementary Fig. 14). Interestingly, the scanning electron microscopy (SEM) results revealed similar microstructures, with only slight difference of ultrasmall porosity profiles as observed in the freeze-dried printed constructs before and after enzymatic digestion (Fig. 3e). Note that SEM images do not directly reveal the interchain distances due to the possible packing of polymer chains during the freeze-drying process. Further porosity measurements via a nitrogen-adsorption method determined that the specific surface area of the Hase-digested samples increased when compared with the untreated controls (Supplementary Table 3), which was resulted from the additional pores generated after cleaving the HA chains. However, the average pore size was slightly reduced after Hase digestion, suggesting that the newly formed pores by molecular cleavage were indeed, small in scale that caused such a reduction in the average size of all the pores present in the system, to a good extent consistent with SEM observations that microscale porosities remained largely unchanged. Our findings led us to conclude that the GelMA/HAMA bioinks with highly tunable stiffness values (~1–180 kPa demonstrated but likely beyond) yielded by post-printing enzymatic digestion, enable facile DLP printing of structurally sophisticated constructs while allowing recapitulation of soft-organ mechanical properties otherwise previously almost impossible.

We have now introduced the concept of molecularly cleavable bioinks as a methodology for 3D soft-tissue bioprinting and studied the possibilities with a representative collection of bioinks based on GelMA/HAMA, followed by the treatment with an enzyme specifically digesting the HAMA component. This method was further expanded to GelMA-cleavable GelMA/HAMA bioinks through digesting GelMA chains using collagenase^45^. Bioinks of GelMA/HAMA (2.5%/1.5%, *M*_w_ of HAMA = 100 kDa) and GelMA/HAMA (2.5%/2.0%%, *M*_w_ of HAMA = 100 kDa) were taken as examples to demonstrate the feasibility of digesting GelMA with collagenase and obtaining softer, HAMA-rich hydrogels. As can be seen from Supplementary Fig. 15, the compressive moduli of the GelMA/HAMA (2.5%/1.5%) constructs were decreased from 26.2 ± 4.2, to 8.2 ± 0.6 and 4.7 ± 1.1 kPa by the treatment of 1- and 2- U mL^−1^ collagenase, respectively, for 24 h. The same tendency could be found when the HAMA concentration was increased to 2.0%, while the impact on lowering hydrogel stiffness was not as significant as the GelMA/HAMA (2.5%/1.5%) constructs. Therefore, the alternative method of cleaving GelMA molecules from GelMA/HAMA-printed constructs also provided an option for lowering hydrogel stiffness through post-printing digestion. Other polymers, which can provide sufficient mechanical properties for bioprinting and be molecularly cleaved by specific enzymes, are possible options to serve as molecularly cleavable bioinks as well. The examples include but are not limited to photocrosslinkable moiety-functionalized polysaccharides, such as dextran and chitosan, as well as glycosaminoglycans, such as chondroitin sulfate and heparin.

### Tissue-fabrication and evaluations

As discussed, our unique strategy of post-bioprinting enzymatic digestion is motivated by the oftentimes contradicting requirements of both structural complexity and biological properties of these fabricated tissue-mimics, such as supporting the growth of cells originated from soft tissues to ensure proper functions. This is particularly problematic in the conventional DLP bioprinting method, which allows bioprinting of sophisticated patterns but only when the mechanics of the hydrogels are sufficiently high^11^. To demonstrate the feasibility of our strategy in soft-tissue bioprinting using DLP, we first sought to evaluate the influence of the Hase enzyme on behaviors of multiple cell types. The results from Supplementary Fig. 16a–c revealed that high enzyme concentrations and long treatment times had negative impacts on the survival and metabolic activities of NIH/3T3 fibroblasts cultured on the surfaces of well-plates. For this reason, NIH/3T3 fibroblasts were treated with lower enzyme concentrations (below 1000 U mL^−1^) for 24 h. Neither cell survival nor metabolic activities were significantly affected under these conditions (Supplementary Fig. 16d–e).

We further assessed the C2C12 myoblast behaviors within the GelMA/HAMA constructs after 1-day digestion by different Hase concentrations (Supplementary Fig. 17). An interesting finding was that the C2C12 myoblasts started to spread on day 3 of culture after 500 U mL^−1^ of enzymatic digestion, which was in line with the cell morphology observed in GelMA (7.5%)-only control. Of note, the latter samples were fabricated through casing since GelMA alone at this concentration was not printable in 3D (Supplementary Fig. 2). Cell spreading within the GelMA/HAMA constructs was particularly attractive as the enzyme concentration was increased to 1000 U mL^−1^. In these constructs, adding HAMA to GelMA followed by Hase digestion showed no negative effect on cell spreading. Hase cleaved interconnected HAMA chains, and thus provided more space for cell growth and spreading.

Considering that the encapsulated cells might leach out during HAMA digestion, we next explored the cell motility using the NIH/3T3 fibroblasts bioprinted in GelMA/HAMA constructs. While a small population of cells (16.0 ± 2.2%) did leach out when the bioprinted samples were treated with Hase (1000 U mL^−1^) for 24 h (Supplementary Fig. 18a, b), the metabolic activities of the cells within the Hase-treated constructs over the subsequent culture period dramatically increased compared to the untreated controls (Supplementary Fig. 18c). On the basis of cell morphological enhancements after enzymatic digestion, we then illuminated the great potential of the DLP-bioprinted GelMA/HAMA constructs followed by enzymatic digestion in the fabrication of biologically relevant tissue-mimics.

#### Bioprinting of skeletal muscle tissue

**Fig. 4:**
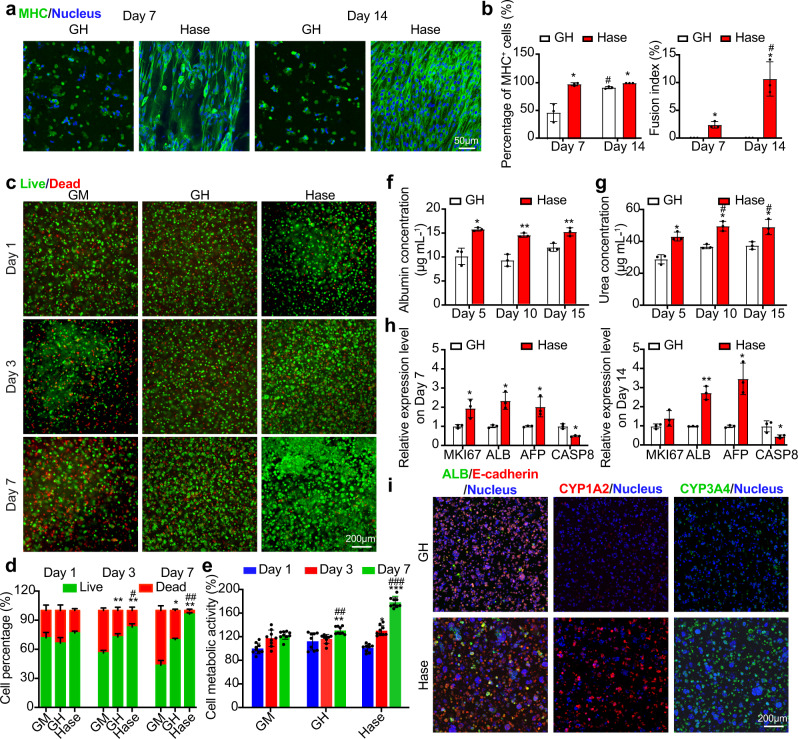
Post-bioprinting enzymatic digestion enhances spreading and functions of skeletal muscle and hepatic cells. **a** Micrographs showing MHC (green) staining of C2C12 cells cultured in the bioprinted constructs from GelMA/HAMA (7.5%/1.5%) without or with Hase digestion (1000 U mL^−1^, 24 h) at the days 7 and 14 after myogenic differentiation. **b** Corresponding quantitative results of the MHC^+^ cells and fusion index. **c** Micrographs showing live (green)/dead (red) staining of HepG2/C3A cells encapsulated in constructs bioprinted from 10% GelMA, and GelMA/HAMA (5.0%/1.5%) without or with Hase digestion (1000 U mL^−1^, 24 h) at the days 1, 3, and 7 of culture. **d** Corresponding quantitative analyses of the percentages of live/dead cells. **e** Quantitative results of MTS assay showing metabolic activities of the HepG2/C3A cells. **f**, **g** Quantitative results of ALB- and urea-secretion levels of the HepG2/C3A cells grown in the bioprinted GelMA/HAMA (5.0%/1.5%) constructs without or with Hase digestion (1000 U mL^−1^, 24 h) after 5, 10, and 15 days. **h** Gene expression profiles showing expression levels of *MKI67*, *ALB*, *AFP*, and *CASP8* for the HepG2/C3A cells grown in the bioprinted GelMA/HAMA (5.0%/1.5%) constructs without or with Hase digestion (1000 U mL^−1^, 24 h) after 7 and 14 days. All gene expression fold changes are relative to the corresponding expressions of the GH group. **i** Confocal immunofluorescence images showing staining for ALB (green) and E-cadherin (red), or CYP1A2 (red), or CYP3A4 (green), with nuclei counterstaining (blue) of the cells cultured in the bioprinted GelMA/HAMA (5.0%/1.5%) constructs without or with Hase digestion (1000 U mL^−1^, 24 h) after 14 days. Images are representatives of *n* = 3 independent experiments. **a** and **b**, **c** and **d**, and **f**–**h**, *n* = 3; **e** *n* = 9; one-way ANOVA; *p < 0.05, **p < 0.01, ***p < 0.001 (**b** and **f**–**h**, compared with the group of GH; **d** and **e**, compared with the group of GM); ^#^*p* < 0.05, ^##^*p* < 0.01, ^###^*p* < 0.001 (**b**, compared with the corresponding results of day 7 in the same group; **d** and **e**, compared with the group of GH; **g** compared with the corresponding results of day 5 in the same group). Data are presented as mean values ± SDs. GM indicates the hydrogel made of 10% GelMA, GH is composed of GelMA/HAMA (7.5%/1.5% for skeletal muscle tissue, 5.0%/1.5% for hepatic tissue), and Hase is the group of GelMA/HAMA treated with Hase (1000 U mL^−1^) for 24 h. MHC myosin skeletal heavy chain, ALB albumin. Source data are provided as a Source Data file.

The first tissue we mimicked was the skeletal muscle, where we bioprinted C2C12 myoblast-embedded GelMA/HAMA (GH group, 7.5%/1.5%) bioink, and further digested the bioprinted muscular tissue in Hase at the concentration of 1000 U mL^−1^ (Hase group) for 24 h. The C2C12 myoblasts cultured in the GH group and the Hase group were evaluated by immunofluorescence staining of myosin skeletal heavy chain (MHC), which is a myogenic differentiation marker^46^. As shown in Fig. 4a, b, the cells displayed an isolated growth pattern with limited cell spreading, as well as impaired myogenic biomarker expression in the GH group after both 7 days and 14 days of differentiation (day 10 and day 17 after Hase digestion). By contrast, the same cells in the Hase-digested samples started to fuse with surrounding cells and self-organized to form myotubes that exhibited spontaneous alignments in most if not all the regions (Fig. 4a, b, Supplementary Fig. 19). Cell fusion and myotube-formation became more significant after 14 days of differentiation, as also quantified by the fusion index plot. From the results we obtained, it was clear that the Hase-digested hydrogels effectively supported C2C12 cell spreading and this cleaved microenvironment favored the differentiation of the cells.

Myogenesis is a process of myoblast differentiation from single cells into multinucleated muscle fibers^47^. During this process, myoblasts recast spatial cellular arrangement over distances without a central coordinator to become well-ordered and multinucleated myotubes from a disordered state of individual, undifferentiated cells. Many rationales behind this process are still unclear; however, researchers have identified several physical factors to play an important role in guiding myoblasts self-organization. For example, it was revealed that C2C12 myoblasts spontaneously assembled into highly aligned myotube tissues when cultured on sufficiently soft yet fully isotropic gelatin substrates^48^. In our study, the enzymatic digestion process resulted in softer hydrogels matching the muscle tissue stiffness. The formed myotubes were likely influenced by the proper hydrogel stiffness and were observed to align only in the Hase-digested samples, which were consistent with the observations from the literature^48^.

#### Bioprinting of hepatic tissue

To create a hepatic tissue model that mimics the native mechanical properties and cellular functions, we encapsulated hepatocyte-like HepG2/C3A cells into the bioprinted GelMA/HAMA (5%/1.5%) constructs followed by post-bioprinting enzymatic digestion (1000 U mL^−1^ for 24 h, Hase group). We specifically compared the culture of HepG2/C3A cells within the Hase group to that before digestion (GH group), and 10% GelMA alone (GM group). The hydrogels made by 5% GelMA have been commonly adopted as the scaffolds for liver tissue engineering^19,49,50^. Hence, we also obtained the 5% GelMA-alone group encapsulating HepG2/C3A cells through the casting method instead of DLP bioprinting, since the bioprinting failed when the GelMA concentration was decreased to under 10% (Supplementary Fig. 2). The live/dead assay illustrated that an obviously increasing level of live cells in the Hase-digested GelMA/HAMA constructs was observed across the 7 days of culture, whereas a constantly decreasing number of live cells could be found in the GM samples at days 3 and 7 (Fig. 4c, d). HepG2/C3A cellular aggregation in the Hase group started at day 3 after enzymatic digestion, with increasing aggregation size over the entire culture period. At day 7, many contiguous cellular aggregates merged together. The results were consistent with those of the cast 5% GelMA group, where the cellular aggregation enlarged throughout the same culture period (Supplementary Fig. 20). The cells in the Hase group were still highly viable and spread well even when cultured for 14 days (Supplementary Fig. 21), suggesting the enhanced cytocompatibility of our post-bioprinting digestion method. On the contrary, the live HepG2/C3A cells in the GH group exhibited slight increase over 7 days of culture, and only a few small aggregates were formed by the cells cultured in the GM and GH constructs (Fig. 4c, d). A similar trend was observed when the cells were cultured for up to 14 days (Supplementary Fig. 21). Metabolic activity assays demonstrated similar cellular responses (Fig. 4e and Supplementary Fig. 20) with the results from the live/dead assay. Hase-digested and 5% GelMA-cast structures presented excellent cell proliferations from 1 to 7 days; however, cells cultured in the GM and GH groups did not significantly improve over the period. These outcomes were consistent with the observations from previous studies, where cells cultured in GelMA at a higher concentration (10%) generally show limited activities^13,16^.

The secretions of albumin (ALB) and urea, which are important indicators of hepatic functions^51^, were further illustrated at higher productions by the encapsulated HepG2/C3A cells in the Hase group, when compared to the GH samples at the 5-day, 10-day, and 15-day time points (Fig. 4f, g). This observation implied the dramatic influence on functional maintenance of hepatic homeostasis with post-treatment of Hase. To further verify this hypothesis, the related gene expressions of cell proliferation markers (*MKI67*), liver function markers (*ALB* and alpha fetoprotein (*AFP*)), and apoptosis markers (caspase 8, *CASP8*) were investigated at days 7 and 14 of culture after Hase digestion. Significant upregulations of the *MKI67*, *ALB*, *AFP* genes along with a lower expression of the apoptosis marker *CASP8* were found in HepG2/C3A cells cultured in the Hase group at both time points (Fig. 4h). Confocal images of the cells cultured in samples showed positive staining for both ALB and E-cadherin (Fig. 4i). Nevertheless, more than 2-fold and 6-fold increases of ALB and E-cadherin expressions, respectively, were recorded in the Hase-digested constructs **(**Supplementary Fig. 22), confirming that post-bioprinting digestion was of paramount importance in terms of supporting ALB-production and epithelial cell junction-formation of these hepatic cells. Encouraged by the observed functional enhancements, we proceeded to evaluate the cytochrome P450 (CYP) enzymes, which are a key enzyme family in liver metabolism defining detoxification and bioactivation processes^52^. As Fig. 4i and Supplementary Fig. 22 indicated, both CYP1A2 and CYP3A4 presented higher expressions in the Hase group compared to the GH group, which displayed potentially enhanced the capabilities of metabolism and drug clearance. It is also worthwhile to mention that the higher CYP3A4 expression itself represents a maturation towards adult hepatocytes as fetal hepatocytes express a relatively low level of CYP3A4^19^.

### Bioprinting of functional mini-brain tissues

The brain is the most complex yet softest organ in the body^53^. Recapitulating the native physicochemical microenvironment in support of neurons and glial cells in vitro is of paramount importance for their proliferation, differentiation, and functionalities^54^. Nevertheless, the fabrication of low-stiffness, truly volumetric brain microtissues using 3D bioprinting has not yet been effectively realized, again because of the contradicting requirements of the ultra-low mechanical properties of bioinks and the structural fidelity, which is a major obstacle in conventional DLP bioprinting. Having demonstrated the ability to bioprint sophisticated 3D structures using our GelMA/HAMA bioink and to mimic the mechanical properties of soft tissues by subsequently digesting the HAMA component, we next proceeded to study the feasibility of this unique strategy in bioprinting ultrasoft tissues such as the brain and supporting the behaviors of encapsulated neural progenitor cells (NPCs).

**Fig. 5:**
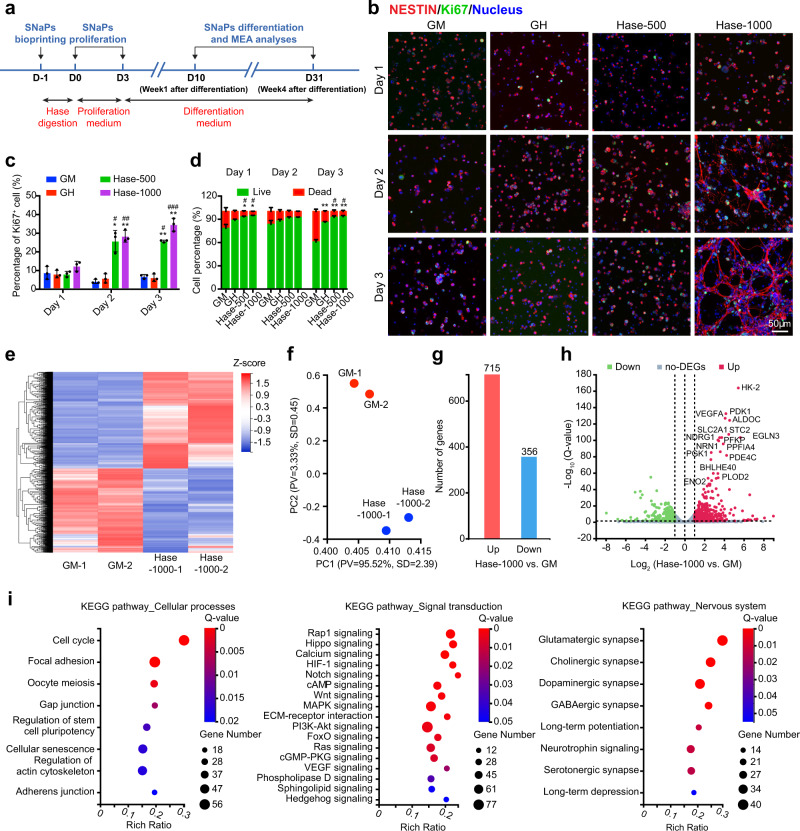
Post-bioprinting enzymatic digestion modulates the activities of SNaPs and facilitates formation of brain-like tissue. **a** Timeline of 3D bioprinting of SNaPs and their subsequent culture protocols. The bioink was GelMA/HAMA (2.5%/1.5%), followed by 500 or 1000 U mL^−1^ of Hase digestion for 24 h post-bioprinting. **b** Fluorescence micrographs of SNaPs cultured in GM and GH constructs without or with Hase digestion (500 or 1000 U mL^−1^, 24 h), stained for NESTIN (red), Ki67 (green), and nuclei counterstaining (blue) at days 1, 2, and 3 after digestion. **c** Corresponding quantitative analyses of the percentages of Ki67^+^ nuclei in the four groups. **d** Quantitative analyses of the percentages of live/dead cells obtained from live/dead staining images. **e** Gene expression clusters for SNaPs after 4 weeks of differentiation (31 days after digestion) in GM and Hase-1000 digested constructs. **f** PCA of gene expression values derived from whole-transcriptome sequencing data for SNaPs after 4 weeks of differentiation in GM and Hase-1000 digested constructs. **g** Total gene numbers of upregulation and downregulation for SNaPs after 4 weeks of differentiation in GM and Hase-1000 digested constructs. **h** Volcano plot of transcriptional landscape comparing SNaPs after 4 weeks of differentiation in GM and Hase-1000 digested constructs. **i** K-means clusters of genes of the KEGG enrichment analyses for SNaPs after 4 weeks of differentiation in GM and Hase-1000 digested constructs. **b** and **c**, and **d** *n* = 3; one-way ANOVA; *p < 0.05, **p < 0.01(compared with the group of GM), ^#^*p* < 0.05, ^##^*p* < 0.01, ^###^*p* < 0.001 (compared with the corresponding results of day 1 in the same group). Data are presented as mean values ± SDs. GM indicates the hydrogel made of 10% GelMA. GH is composed of GelMA/HAMA (2.5%/1.5% for brain-like tissue), and Hase-500 and Hase-1000 are the groups of GelMA/HAMA treated with Hase (500 and 1000 U mL^−1^, respectively) for 24 h. Source data are provided as a Source Data file.

To achieve a robust and rapid generation of neuronal cells, we used human stem cell-derived neural progenitors as the seeding cell for bioprinting and subsequently differentiated them into neuronal cells post-bioprinting. Transcription factor-programming with the lineage-specific transcription factors emerges as a strategy for the induction of stem cell differentiation^55^. Neurogenin-2 (NGN2), a transcription factor related to neuronal development, has been reported to produce Stem cell-derived NGN2-accelerated Progenitor cells (SNaPs) from human stem cell-derived NPCs^56^. The generated SNaPs are multipotent cells, which are capable of differentiating into glia and neurons. The DLP-bioprinted SNaPs encapsulated in 10% GelMA (GM group), 2.5%/1.5% GelMA/HAMA (GH group), and 2.5%/1.5% GelMA/HAMA with Hase digestion (500 U mL^−1^ for 24 h, Hase-500 group; or 1000 U mL^−1^ for 24 h, Hase-1000 group), in the shape shown in Fig. 3e, were cultured in the proliferation medium for 3 days after Hase digestion, followed by neuronal induction in the differentiation medium (Fig. 5a). First, we investigated whether the Hase digestion of the GelMA/HAMA constructs influenced the proliferation and survival of SNaPs. Compared with the control groups of GM and GH, we found that the proliferation of SNaPs, indicated by the percentage of Ki67^+^ cells, increased significantly in the groups treated with Hase (Hase-500 and Hase-1000) at day 2 and day 3 after digestion (Fig. 5b, c). Additionally, the live/dead staining was conducted to evaluate SNaP viability before and after digestion. As shown in Fig. 5d and Supplementary Fig. 23, the viability of SNaPs increased to 93.0 ± 1.0% or 94.7 ± 0.46% and 92.3 ± 2.08% or 93.3 ± 1.53% at day 1 and day 3 after digestion by 500 U mL^−1^ or 1000 U mL^−1^ of Hase, respectively. These results elucidated that the post-bioprinting enzymatic treatment of GelMA/HAMA constructs facilitated the proliferation and viability of SNaPs.

#### Transcriptomic analyses

Global transcriptome profiling was conducted through RNA sequencing (RNA-seq) on SNaPs after 4 weeks of differentiation (or 31 days after digestion) in the GM and Hase-1000 samples. No non-digested GH samples were compared since most cells were already dead at this time point of culture not allowing RNA-extraction. The heatmap presented differentially expressed genes (DEGs) between Hase-1000 and GM samples (Fig. 5e). The principal components analysis (PCA) implied that the GM group had a drastic transcriptional profile difference with the Hase-1000 samples (Fig. 5f). Overall, 715 genes were significantly upregulated while 256 genes were dramatically downregulated (*Q*-value < 0.05, Fig. 5g). As shown in the volcano plot of Fig. 5h, the overexpressions of hexokinase 2 (*HK-2*) and phosphoinositide-dependent protein kinase 1 (*PDK1*) were found in the Hase-1000 group compared to the GM group, suggesting inhibited apoptosis and accelerated cell proliferation after Hase digestion^57,58^. Moreover, neuronal-related genes were upregulated in the Hase-1000 group, such as stanniocalcin 2 (*STC2*), which is an injury-responsive gene required for axon regeneration;^59^ neuritin 1 (*NRN1*), which encodes extracellular glycosylphosphatidylinositol (GPI)-anchored protein to stimulate axonal plasticity, dendritic arborization, and synapse maturation;^60^ and enolase 2 (*ENO2*)^61^, which exhibits neurotrophic and neuroprotective properties in the central nervous system (CNS)^62^.

To elucidate the biological processes within the different bioink formulations and treatment conditions, we performed both Gene Ontology (GO) and Kyoto Encyclopedia of Genes and Genomes (KEGG) pathway-enrichment analyses with the RNA-seq results of GM and Hase-1000 samples. The differences of transcriptional profiles in molecular functions and biological processes are displayed in Fig. 5i. Positive cell cycle regulation indicated an enhanced proliferation of SNaPs in the Hase-1000 group compared to the GM group, which was consistent with the SNaP proliferation results. Cellular processes related pathways including focal adhesion, gap junction, regulation of actin cytoskeleton, and adherens junctions were better-represented in the Hase-1000 samples compared to the GM samples. Modulation by these pathways would enhance cell adhesion, cell–cell interactions, and cell proliferation. Therefore, our transcriptome analyses revealed that the Hase-digestion process provided extra matrix spaces and thus improved the microenvironment for SNaP growth, maturation, and function.

We further focused on several mechanotransduction pathways to better understand the major differences for SNaP behaviors within GM and Hase-1000 groups. Among the enriched pathways, the hippo has been demonstrated as an essential signaling pathway in the survival and differentiation of neural stem cells (Fig. 5i)^63^, activated by mechanical cues generated from altered cell shape, cell polarity, cell–cell junctions, or extracellular matrix (ECM) stiffness. During tissue regeneration or organ development, cells constantly respond to mechanical stress from neighboring cells and ECM, or shear force when they migrate^64^. These factors are transmitted via membrane receptors, actin cytoskeleton, and the nuclear membrane to influence gene expressions within the nuclei, resulting in changes not only in cell morphology and survival but also in cell fate-specification^65^. More importantly, the highly enriched terms related to the nervous system (Fig. 5i) illuminated that intended cell differentiation clearly happened more efficiently in the Hase-1000 samples. Specifically, a heatmap was produced to compare DEGs involved in the glutamatergic synapse pathway, revealing that genes *GNB5*, *GNG7*, *ADCY8*, *GNG4*, *SLC17A7*, *MAPK3*, *GRIN3B*, *CACNA1A*, *ADCY5*, *GRIN1*, *DLG4*, *PRKCB*, *GRIK5*, *GLS2*, *PRKCG*, *ADCY7*, *GNG13*, *ADCY3*, *KCNJ3*, and *GRIN2D* were upregulated in the Hase-1000 group (Supplementary Fig. 24). Gene set enrichment analysis (GSEA) results also elucidated that compared to the GM group, the Hase-1000 samples expressed enriched gene sets involved in synaptic signaling, neurotransmitter transport, and long-term synaptic potential pathways.

#### Immunological staining

**Fig. 6:**
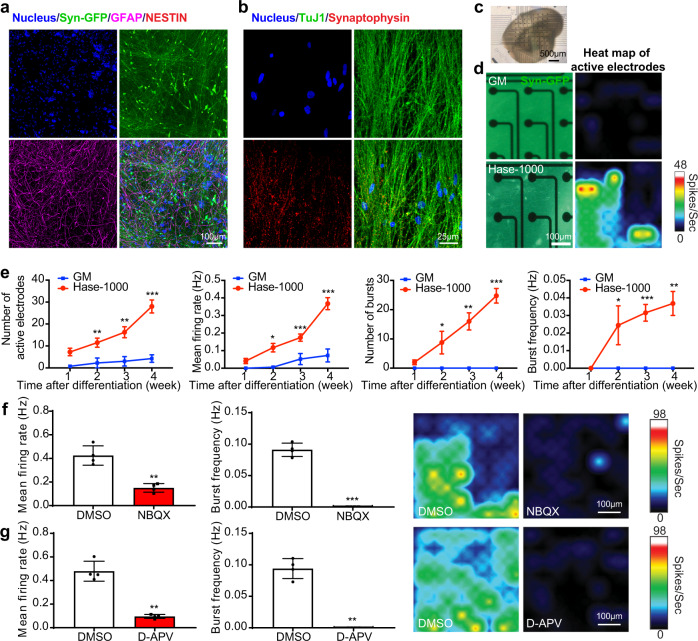
Differentiation and functionalities of bioprinted SNaPs of brain-like tissue with the GelMA/HAMA bioink following enzymatic digestion. **a**, **b** Confocal fluorescence micrographs showing immunostaining results of (**a**) protein markers representing neurons (SNaPs, green), astrocytes (GFAP, magenta), and neural progenitor cells (NESTIN, red) after 4 weeks of differentiation; and **b** neuronal markers including neurites (TUJ1, green) and synaptic vesicle proteins (synaptophysin, red). In all cases, the bioink was GelMA/HAMA (2.5%/1.5%), followed by 1000 U mL^−1^ of Hase digestion for 24 h post-bioprinting. **c** Optical image showing the 4-week differentiated mini-brain placed on top of the MEA, with maintenance of the overall bioprinted brain shape similar to that shown in Fig. 3e. **d** High-magnification fluorescence micrographs showing SNaPs after 4 weeks of differentiation directly in contact with the MEA surface and representative heatmaps of active electrodes. **e** Electrophysiological properties of SNaP-derived neurons in the bioprinted mini-brains with 10% GelMA and GelMA/HAMA (2.5%/1.5%), followed by 1000 U mL^−1^ of Hase digestion for 24 h post-bioprinting at 1–4 weeks after differentiation: the percentages of active MEA electrode plateaus; the mean firing rates; the numbers of bursts; and the network burst frequencies. *n* = 4; one-way ANOVA; *p < 0.05, **p < 0.01, ***p < 0.001 (compared with the group of GM). Data are presented as mean values ± SDs. **f**, **g** Electrophysiological property changes of SNaP-derived neurons in the bioprinted mini-brains post-enzymatic digestion, without or with treatment of (**f**) AMPA receptor-antagonist NBQX (10 µM) or (**g**) NMDA receptor-antagonist D-APV (50 µM) at 4 weeks after culturing in the MEA plates. *n* = 4; two-tailed student’s *t*-test; *p < 0.05, **p < 0.01, ***p < 0.001 (compared with the group of DMSO). Data are presented as mean values ± SDs. GM indicates the hydrogel made of 10% GelMA. Hase-1000 indicates the groups of GelMA/HAMA treated with Hase (1000 U mL^−1^) for 24 h. Source data are provided as a Source Data file.

The ability to differentiate into multiple cell types of the neural and glial lineages is a hallmark of NPCs^66^. To assess the differentiation capacities of SNaPs, we stained SNaPs in the bioprinted constructs for glial fibrillary acidic protein (GFAP), which is expressed by both immature and mature astrocytes as well as NPCs^67^, and nestin, as an immature neural marker^68^, during the process of differentiation. Immunostaining of 4-week-differentiated SNaPs (or 31 days after digestion) within the constructs presented the expression of neuronal marker (Syn-green fluorescent protein, GFP) and GFAP, but NESTIN^+^ cells were not detected (Fig. 6a), indicating the multipotency of the bioprinted SNaPs achieved through digestion of the GelMA/HAMA hydrogel. To distinguish between astrocytic differentiation and remaining in a precursor state, the S100 calcium binding protein B (S100B) staining was performed on the Hase-1000 sample to confirm the astrocytic differentiation of the bioprinted SNaPs within the Hase-digested GelMA/HAMA constructs (Supplementary Fig. 25)^69^. Through staining of neuron-specific class III *β*-tubulin (Tuj1) and synaptophysin, we observed the characteristic punctate expression of the synaptic marker synaptophysin (Fig. 6b), which revealed that the bioprinted SNaPs were capable of differentiating into neurons and displaying functional synaptic proteins. Our findings suggested that these 3D-bioprinted SNaP-encapsulating constructs followed by post-bioprinting enzymatic digestion could enable the cells to differentiate into neurons and astrocytes, which are representative cell types of neuronal tissues.

Another promising finding was that the different matrix rigidities had a significant influence on SNaP cell differentiation. Immunostaining of 3D-bioprinted SNaPs showed that Hase-1000 inducted formation of both neurons (Syn-GFP) and astrocytes (GFAP) (Supplementary Fig. 26), whereas only neurons were observed in the Hase-500 samples. The mechanical properties of the bioprinted constructs treated by Hase-500 and Hase-1000 were 3.98 kPa and 1.29 kPa, respectively, representing a notable difference in matrix rigidity. Different GelMA/HAMA formulations also exhibited apparent variations in subtype-specification after differentiation (Supplementary Fig. 27). Both 5.0% and 2.5% GelMA bioinks homogeneously mixed with 1.5% HAMA after 1000 U mL^−1^ Hase digestion showed both astrocyte- and neuron-formation. By contrast, SNaPs bioprinted by the GelMA/HAMA (2.5%/3.0%) bioink possessing a relatively high stiffness after Hase digestion, exhibited the limited cell spreading and neuronal differentiation. These results tied well with previous studies wherein the mechanotransduction of neuronal cells, defined as the conversion of force a cell generates through cell-substrate bonds to a chemical signal, has great significance on neural tissue engineering^70,71^. Our findings confirmed that matrix rigidity is an essential biophysical cue affecting neuronal induction and subtype-specification, and suggested that 3D-bioprinted brain-mimics with native tissue-matching mechanical properties could thus serve as a promising platform for nervous system modeling.

#### Electrophysiology assessments

To access the electrophysiological functions of the SNaP-derived neurons in our bioprinted 3D mini-brains, multi-electrode array (MEA) was used to detect action potentials and validate the formation of functional synaptic networks^72,73^. Neurons in Hase-1000 and GM samples were separately placed on the electrode arrays of MEA plates and the electrophysiological signals were recorded every week for up to 4 weeks after differentiation (Fig. 6c–e). Also note again, the readily maintained overall shape of the bioprinted mini-brain after digestion and culture, shown in Fig. 6c. The average number of active electrodes increased over time and was higher in neurons of the Hase-1000 group than in those of the GM group (Fig. 6d–e), indicating the increased number of active neurons in the Hase-1000 samples. The mean firing rate followed a similar time course-dependent increase and was significantly elevated in neurons in the Hase-1000 samples. Burst analyses of the MEA recordings identified synchronous firing events as bursts with a burst frequency detectable from as early as week 2. Notably, the number of bursts and burst frequency increased with time in the Hase-1000 samples, but neurons within the GM group showed negligible burst events, confirming the inability of neurons to form synaptic networks in the GM hydrogel.

We finally confirmed the synaptic contributions to burst activities in the Hase-1000 samples at week 4 after culture onto the MEA plates through pharmacological interventions. The mean firing rate and burst frequency were significantly reduced by 2,3-dihydroxy-6-nitro-7-sulfamoyl-benzo(F)quinoxaline (NBQX, Fig. 6f), an *α*-amino-3-hydroxy-5-methyl-4-isoxazole-propionate (AMPA) receptor-antagonist^74^, suggesting that the neuronal network activity by the cells in the Hase-1000 group was driven in part by excitatory synaptic transmission mediated by ionotropic glutamate receptors. Similarly, the addition of D-2-amino-5-phosphonovaleric acid (D-APV), an N-methyl-D-aspartate (NMDA) receptor-antagonist^75^, significantly reduced the firing and burst frequency (Fig. 6g). Our electrophysiological results demonstrated that the Hase-1000 treatment promoted the synaptic activity and network formation of SNaP-induced neurons, which was consistent with our transcriptomic profiling data as well as related analyses. Overall, the difference in gene expression and subsequent functional changes of cells differentiated in the post-bioprinting enzymatically digested mini-brains compared to the control were likely combined results of the dimensionality and tunable mechanical cues of the ECM-like matrix, as well as favored cellular crosstalk of SNaPs.

Collectively, we observed from our series of studies that the presence of HAMA, although strengthening printability, might pose unwanted negative impacts on cellular growth and functions especially when soft tissues are involved, since addition of HAMA would inevitably result in densely crosslinked hydrogel networks. We hence proposed that rational utilization of Hase could cleave the HAMA molecules in the bioprinted HAMA/GelMA constructs and reduce the crosslinking densities of the entire hydrogel networks. As a result, mechanical stiffness of the bioprinted constructs could be precisely tuned, where the cleaved HAMA also provided cells with more space to grow and favored their functions, such as stem cell differentiations otherwise not readily attainable in similar hydrogel systems.

In conclusion, we report the utilization of a GelMA/HAMA bioink as a generalized methodology, which may transform the field of DLP bioprinting. This approach offers unprecedented bioprinting possibilities where designed constructs could be printed at high structural sophistication and fidelity, and post-printing enzymatically digested for the HAMA component to meet mechanical and biological requirements of target tissues. These two requirements are usually contradictory posing a major challenge in conventional DLP-based bioprinting, which is successfully resolved using our extremely simple yet powerful molecular cleavage approach. We have established an extensive library of mechanical parameter maps based on both experimental results and theoretical modeling, resulting in broadly tunable and precisely controllable mechanical properties by adjusting bioink formulations and digestion conditions.

Our demonstration of this enabling DLP-bioprinting strategy presented the excellent printability of tissue-mimicking structures featuring sophisticated shapes and internal architectures, which were well-retained after enzymatic digestion. Importantly, our results provided evidence of cell morphological and functional enhancements of encapsulated cells, suggesting the suitability of this method for the bioprinting of living constructs of soft-tissue origins, such as the brain, liver, and muscles. Of special interest, as illuminated by immunocytochemistry and RNA-seq data, SNaPs in our DLP-bioprinted volumetric mini-brain tissues differentiated into neurons and astrocytes; these neurons exhibited strong electrophysiological and other brain-like properties, which are extremely hard to achieve before. The broad implication of the present research lies in expanding the palette of bioinks usable for high-fidelity DLP bioprinting, enabling the biofabrication of mechanically tunable constructs to meet the biological function requirements of target tissue-mimics, in particular those that are soft and ultrasoft. Our technology does not come without limitations. For example, the careful balance between enzymatic digestion conditions and cellular viability would need to be considered when applying it to practice, among others. With further optimizations, this unique molecular cleavage strategy relying on post-bioprinting enzymatic treatment is anticipated to find widespread applications in tissue and tissue model engineering.

## Methods

### GelMA synthesis and characterizations

GelMA was synthesized according to previously reported methods^76,77^. In brief, type A gelatin from cold-water fish skin (10 g, *M*_w_ = 60 kDa, Sigma-Aldrich, USA) was dissolved in phosphate-buffered saline (100 mL, PBS, ThermoFisher, USA) at the concentration of 10%. Methacrylate anhydride (12 mL, MA, Sigma-Aldrich) was then added dropwise and was reacted with gelatin solution at 50 °C for 2 h. After diluting the solution 1:1 with warm PBS (40 °C) to stop the reaction, dialysis was conducted for 7 days against deionized (DI) water using dialysis membrane (*M*_w_ cut off (MWCO): 12–14 kDa, Spectrum Chemical, USA) at 40 °C to remove low-molecular weight impurities. Subsequently, the solution was lyophilized and stored at −20 °C in dark until use. The lyophilized GelMA was further characterized by ^1^H NMR spectroscopy (Bruker Avance II 300-MHz NMR, USA). NMR data were processed with the MestreNova (6.2.0) software. The primary amine contents of gelatin and synthesized GelMA were evaluated by TNBSA assay^78^.

### HAMA synthesis and characterizations

The synthesis of HAMA followed the procedure as previously described^79^. Briefly, 4.0 g of HA with different *M*_w_ ranging from 10 to 1500 kDa (10, 60, 100, 200, 500, or 1,500 kDa, HAworks, USA), was fully dissolved in 200 mL of DI water at 4 °C. 133.3 mL of dimethylformamide (DMF, Sigma-Aldrich) and 7.88 mL of MA were added into HA solution under vigorous stirring. The pH of the solution was regulated to pH 8–9 with 1-M sodium hydroxide (Sigma-Aldrich) solution. The reaction was kept at 4 °C under continuous stirring for another 18 h. Subsequently, 0.5-M NaCl (Sigma-Aldrich) was dissolved in the mixture, and the mixture was precipitated in a doubled volume of ethanol (Sigma-Aldrich). HAMA was then collected as white pellets after precipitation. The precipitate was washed with ethanol for 3 times before being dissolved in DI water and the solution was dialyzed against DI water for 5 days. The purified product was obtained by lyophilization and characterized by ^1^H NMR spectroscopy.

### FITC-conjugated HAMA (HAMA-FITC) synthesis

To conjugate FITC to HAMA, we first dissolved 1.0 g of HAMA (100 kDa) in 2-(*N*-morpholino)ethanesulfonic acid (MES) buffer (pH = 5.6, Sigma-Aldrich). Then, the 0.1-M *N*-(3-dimethylamino propyl)-*N*-ethylcarbodiimide (EDC, Sigma-Aldrich) and 0.2-M *N*-hydroxysuccinimide (NHS, Sigma-Aldrich) mixture was added to activate the carboxylate groups on HAMA. 10-µg mL^−1^ FITC-poly(ethylene glycol)-amine (FITC-PEG-NH_2_, *M*_w_ = 2 kDa, Nanocs, USA) was added and reacted overnight at 4 °C in dark. The reacted solution was dialyzed against DI water for 5 days and then lyophilized for use.

### DLP-based 3D bioprinter

A DLP-based 3D bioprinter was built in-house using a projection device^80^, the PRO4500 Optical Engine, with a display resolution of 912 × 1140 pixels (Wintech Digital Systems Technology, USA). The optical pattern generated by the projector was reflected by an aluminum front-coated mirror (Edmund Optics, USA) placed at 45° and focused to obtain the field of view of 65.6 × 41 mm^2^ and an *x*–*y* resolution of 50 µm. A build platform hosting a glass slide (Carolina, USA) allowed the printed construct to attach during and after printing, which was controlled by a stepper motor linear drive (TOAUTO, USA) along the *z*-axis. Custom software was developed in MATLAB (v2020, MathWorks, USA) to control the DLP bioprinting processes. A Teflon film (Random Technologies, USA) was used as the bottom of the vat to enable a clear transmission of light and to provide an oxygen-permeable window. Multiple printing models used in this study, including the cube (Fig. 2a), the pyramid (Fig. 2c), the cuboid with a spiral channel (Fig. 2e), the hepatic lobule (Fig. 3e), and the lung (Fig. 3e), were designed in SolidWorks (Dassault Systèmes, France), whereas those of the gyroid (Fig. 2d), the brain (Fig. 3e), and the heart (Fig. 3e), were acquired from Thingiverse (MakerBot Industries, USA) and reused with the permission of their creators. The model of the torus knot in Fig. 2f was obtained from a previous study^11^. Each designed digital file was sliced into a series of two-dimensional (2D) images at a thickness of 100 μm using the open-source DLP slicer (https://formlabs.com/blog/open-source-dlp-slicer/, Formlabs, USA).

### 3D printing procedure

The desired ink was transferred into the vat before printing, followed by lowering the build platform to the initial printing position. The full control over the image projection and the movement of the build plate was realized by our customized software^40^. After printing was completed, the 3D-printed construct was removed from the build platform and washed in PBS multiple times to remove the uncrosslinked ink. For visualizing the internal hollow structures within the printed 3D constructs, we perfused the painting pigment (Easyou, China) solution into the open channels. To demonstrate the possibility of complex printing structures using the enzymatically digestible inks, the ink formulated with GelMA/HAMA (5.0%/3.0%, *M*_w_ = 100 kDa), 1-mM/10-mM tris(2,2-bipyridyl)dichlororuthenium(II) hexahydrate (Ru)/sodium persulfate (SPS) (Advanced BioMatrix, USA), and 2.0% photoabsorber (Ponceau, Sigma-Aldrich) was selected as an example. The pyramid (4 × 4 × 4 mm^3^), the gyroid (8 × 8 × 8 mm^3^), the cuboid containing a spiral channel (10 × 5 × 4 mm^3^), and the torus knot (18-mm length and 5-mm height) were printed with 15 s of exposure time for each layer (100 μm), in 10 min, 20 min, 10 min, and 12.5 min, respectively.

### Measurements of working curves of GelMA/HAMA inks

The working curves of GelMA/HAMA inks were measured following the method as previously reported^40^. A square pattern (4 × 4 mm^2^) was printed on the cover glass using the inks of GelMA/HAMA (5.0%/3.0%) and 1-mM/10-mM Ru/ SPS without or with the photoabsorber (0.0, 1.0, 2.0, and 3.0% Ponceau), under the exposure times between 5 s and 30 s. Optical images of the thicknesses of the printed constructs were captured using a microscope (Nikon, Japan) and measured with ImageJ (v2.0.0-rc-69/1.52p, National Institutes of Health, USA).

### Printability and resolution evaluations of GelMA/HAMA inks

To evaluate bioink printability, HAMA stock solutions with different *M*_w_ were prepared by dissolving into PBS to be 1.0% HAMA (*M*_w_ = 1500 kDa) or 5.0% HAMA (*M*_w_ = 60, 100, 200, or 500 kDa). GelMA was dissolved in PBS as a stock solution of 20%. HAMA and GelMA solutions were mixed into the final formulations of 0.25, 0.50, 0.75, 1.00, 1.25, 1.50, 1.75, 2.00, 2.25, 2.50, 2.75, or 3.00% HAMA, and 2.5, 5.0, or 7.5% GelMA. Additionally, 1-mM/10-mM Ru/SPS and 2.0% photoabsorber were selected based on desired layer thicknesses. To fabricate the 3D cubic construct used to assess the printability, a cube of 4 × 4 × 4 mm^3^ was printed with a 100-μm thickness and a 30-s printing time for each layer.

The printing resolutions of GelMA/HAMA inks were tested using a standardized evaluation model and method as previously reported. A radial pattern formed by ten lines with a central circle design was printed on the cover glass. The diameter of the central circle pattern was derived from the width and the number of lines. Printing resolution (*p*), defined as the distance between two recognizable independent points, can be calculated by Eq. (4):

4 $$p=D\times {{\tan }}\frac{\pi }{n}-\frac{h}{{{\cos }}\frac{\pi }{n}}$$

where *D* is the diameter of the printed central circle measured from the optical image of printed structure, *n* is the number of radial lines, and *h* is the width of the designed lines. The inks of GelMA/HAMA (5.0%/3.0%) and 1-mM/10-mM Ru/ SPS without or with 2.0% photoabsorber were printed under the exposure times between 2.5 s and 30 s. Optical images of the printed constructs were captured using a microscope and measured with ImageJ.

### Measurements of mechanical properties

To measure the mechanical properties of hydrogels made from different inks, the inks were cast in polydimethylsiloxane (PDMS, Dow, USA) molds (4 mm in width, 3 mm in length, and 3 mm in height) and subsequently photocrosslinked. The compressive moduli of GelMA/HAMA constructs before and after Hase (Sigma-Aldrich) digestion were conducted as described before^79^. Compression tests were performed on a 6800 SERIES mechanical tester (Instron, USA) with a ramp of 2.0 N min^−1^ up to a maximum of 100.0 N. Bluehill Universal (v4.13, Instron) and origin (v9.8, OriginLab, USA) were used to analyze the mechanical tests data. The compressive modulus was calculated according to the slope of the stress-strain curve within the linear region between 0 and 20% strain.

### Enzymatic digestion of GelMA/HAMA constructs

HAMA of 100 kDa in *M*_w_ was selected to investigate the effects of Hase digestion on the mechanical properties of printed constructs. The constructs with the same size (4 × 4 × 2 mm^3^) fabricated by 1.5% or 3.0% HAMA and 2.5%, 5.0%, or 7.5% GelMA were immersed in Hase solutions (0, 75, 150, 300, 500, or 1000 U mL^−1^) for different treatment times (0, 1, 2, 4, 8, 12, or 24 h) under 37 °C with shaking. All the digested constructs were measured for their mechanical properties as described above. For the visualization of digestions, 1.5% HAMA-FITC was used to combine with 2.5%, 5.0%, or 7.5% GelMA. The samples were printed onto cover glasses with the same size (4 × 4 × 1 mm^3^) and transfer into the wells of 24-well plates for post-printing enzymatic digestion. The Hase solutions were prepared with different concentrations of 0, 75, 150, 300, 500, and 1000 U mL^−1^, or different digestion durations of 0, 1, 2, 4, 8, 12, and 24 h under 37 °C. Fluorescence micrographs were captured at the corresponding time points, and the fluorescence intensities were analyzed with ImageJ.

The diffusion coefficient of Hase was measured following previous methods^81^. Briefly, hydrogel samples were prepared by photocuring the GelMA/HAMA (5.0%/3.0%) bioink in rectangular cuvettes (2 × 10 × 45 mm^3^). The obtained samples sealed in cuvettes were first treated with PBS and Hase (1000 U mL^−1^) for 24 h at 37 °C. To measure the diffusion coefficients, 2 mg mL^−1^ of FITC-dextran (*M*_w_ = 60 kDa) was added to the top of the cured hydrogel in each cuvette. Then, the fluorescence images of samples were taken after contacting with FITC-dextran for 1 min using a fluorescence microscope (Zeiss, Germany). The fluorescence intensities were analyzed by ImageJ. To normalize the fluorescence intensities, the fluorescence intensity at the opening side of cuvette was set to 1, and the value at the end of cuvette was set to 0. The diffusion through the hydrogel could be described using the following Eq. (5),

5 $$F\propto {erfc}\;\left(\frac{x}{2\sqrt{{D}_{{eff}}t}}\right)$$

where *erfc* is the complementary error function, *F* is the normalized fluorescence intensity, *x* is the distance from the opening side (cm), *t* is the time since the FITC-dextran and hydrogel contact (s), and *D*_*eff*_ is the effective diffusion coefficient (cm^2^ s^−1^).

### Mathematical modeling

The mechanical properties of fabricated constructs were primarily affected by the GelMA concentration (%), the HAMA concentration (%), the Hase concentration (U mL^−1^), and the digestion duration (h). A mathematical model was used to describe and predict the moduli of the constructs based on these parameters. The effects of these parameters on the moduli were represented by the mechanical data from the experiments, which were then fitted with a quadratic function to create response surfaces. The model can then be used to approximate and explore the functional surfaces between response variables and a set of design factors^82^. In this study, we applied regression modeling and optimization methods to the historical data to determine the best model to fit the system.

The compressive modulus (kPa) was correlated with the experimental parameters A: GelMA concentration (%), B: digestion time (h), C: Hase concentration (U mL^−1^), and D: HAMA concentration (%). A second-degree polynomial model Eq. (6) was fitted to the experimental data.

6 $$Y={a}_{0}+{\sum }_{i}{b}_{i}{x}_{i}+{\sum }_{i,j}{c}_{{ij}}{x}_{i}{x}_{j}$$

where *Y* was the predicted response, *x*_*i*_, and *x*_*j*_, were experimental parameters, and *a*_*0*_, *b*_*i*_, and *c*_*ij*_ were constant, linear, and quadratic coefficients, respectively. When *i* ≠ *j*, these were interaction coefficients. The signal-to-noise ratio of the model was quantified by adequate precision, which compared the range of predicted values at the experimental points to average prediction error. Excellent accuracy of >4.0 indicated that the model was suitable to navigate the design space for prediction.

### Collagenase digestion of GelMA in GelMA/HAMA constructs

GelMA/HAMA (2.5%/1.5%, *M*_w_ of HAMA = 100 kDa) and GelMA/HAMA (2.5%/2.0%%, *M*_w_ of HAMA = 100 kDa) were selected to demonstrate the feasibility of digesting GelMA molecules with collagenase and obtaining softer, HAMA-rich hydrogels. The fabricated constructs were immersed in collagenase type II solution (Worthington Biochemical, USA) of 0, 1, and 2 U mL^−1^ for 24 h under 37 °C with shaking. All the digested constructs were measured for their mechanical properties as described above.

### Evaluation of cell leaching

The constructs (cubes of 4 × 4 × 2 mm^3^) were bioprinted with a 400-μm thickness and a 30-s printing time for each layer using the bioink of GelMA/HAMA (5.0%/3.0%) containing NIH/3T3 fibroblasts (6 × 10^6^ cells mL^−1^, CRL-1658, American Type Culture Collection (ATCC), USA). After bioprinting, the samples were transferred into the wells of a 24-well plate for enzymatic digestion in 1000 U mL^−1^ of Hase for 24 h. The images of leached cells in the wells were captured using a microscope and the leached cell numbers were quantified with ImageJ. Moreover, cellular metabolic activities were assessed by the 3-(4,5-dimethylthiazol-2-yl)-5-(3-carboxymethoxyphenyl)-2-(4-sulfophenyl)-2H-tetrazolium (MTS) assay using the CellTiter 96^®^ AQueous Assay (Promega, USA) according to the manufacturer’s instructions. Briefly, the samples were incubated with the MTS assay reagent for 4 h in the dark. Subsequently, the absorbance values were measured at 490 nm with a microplate reader (Molecular Devices, USA).

### Fabrication and characterizations of tissue-mimics

The tissue mimics were printed with selected inks at 15-s exposure time for each layer (100 μm of layer thickness), where 2.5%, 5.0%, 5.0%, or 7.5% GelMA containing 1.5% or 3.0% HAMA, 1-mM/10-mM Ru/SPS, and 2.0% photoabsorber were applied to print the brain-like (8-mm length and 5-mm height), liver-like (8 × 6 × 5 mm^3^), lung-like (6 × 6 × 6 mm^3^), or heart-like (8-mm height) structure, respectively. To achieve tissue-specific mechanical properties, the fabricated constructs were treated with Hase of 1000 U mL^−1^ for 24 h for the brain-, liver-, and heart-mimics, but with Hase of 500 U mL^−1^ for 24 h for the lung-mimic. SEM (Zeiss, Germany) was used to visualize the microstructures of the printed constructs before and after Hase digestion after lyophilization. Zeiss SmartSEM software (v05.07 SP4) was used to acquire SEM images. In addition, the pore size and surface area were further measured by a Brunauer-Emmett-Teller (BET) surface area analyzer (NOVA 2200E, Quantachrome, USA). The data was obtained from nitrogen-adsorption at 77 K. Before adsorption, all samples were prepared by degassing at 80 °C for 48 h. BET surface area and Barrett-Joyner-Halenda (BJH) cumulative pore volume/distribution were measured and analysed by the NOVA software.

### Muscle tissue-bioprinting and characterizations

C2C12 mouse skeletal myoblasts (CRL-1772, ATCC) was cultured in Dulbecco’s modified Eagle medium (DMEM, ThermoFisher) supplemented with 10 v/v% fetal bovine serum (FBS, Gibco, USA) and 1 v/v% antibiotic-antimycotic (Gibco). C2C12 myoblasts (passages 3–6, 6 × 10^6^ cells mL^−1^) were mixed with GelMA/HAMA (7.5%/1.5%) and 1-mM/10-mM Ru/SPS as the bioink. To make the imaging evaluations of bioprinted tissues easier, cubes of 4 × 4 × 2 mm^3^ were bioprinted with a 400-μm thickness and a 30-s printing time for each layer. After bioprinting, the constructs were transferred into the wells of a well plate for enzymatic digestion in 1000 U mL^−1^ of Hase solution for 24 h, and then replaced by complete culture medium.

To induce differentiation, constructs containing C2C12 myoblasts were starved of serum and cultured in DMEM containing 2 v/v% horse serum (ThermoFisher) at 3 days after Hase digestion. The differentiation medium was changed every day to provide enough nutrition for cell growth. At 7 and 14 days after differentiation, the samples were fixed with 4% paraformaldehyde (Sigma-Aldrich) for 15 min, permeabilized with 0.03 v/v% Triton X-100 (Sigma-Aldrich) for 10 min, and then blocked by 5% bovine serum albumin (BSA, Sigma-Aldrich) for 1 h at room temperature. The samples were incubated with the primary antibody of MHC (Abcam, USA, dilution 1:100, cat no. ab91506) overnight at 4 °C, and then incubated with the secondary antibody (Alexa Fluor 488 goat anti-rabbit IgG, dilution 1:500, ThermoFisher, cat no. A32731) at 37 °C for 2 h. 4', 6-diamidino-2-phenylindole (DAPI, Vector Laboratories, USA, dilution 1:2000, cat no. D1306) was finally used to counterstain the nuclei. The stained samples were visualized by a confocal microscope (LSM880, Zeiss). The acquired images from ZEN Black 2.1 (Zeiss) were analyzed with ImageJ. The fusion index was calculated as the ratio of the nucleus number for cells with two or more nuclei to the total number of nuclei.

### Liver tissue-bioprinting and characterizations

We bioprinted the liver tissue-mimic out of bioink compositions of GelMA/HAMA (5.0%/1.5%) or 10% GelMA only (minimally printable concentration of GelMA for DLP in the absence of HAMA), and 1-mM/10-mM Ru/SPS, using 8 × 10^6^ cells mL^−1^ of HepG2/C3A cells (CRL-10741, ATCC). The bioprinted constructs (cubes of 4 × 4 × 2 mm^3^) were transferred into the wells of a well plate, where DMEM containing FBS in the absence or presence of Hase solution at 1000 U mL^−1^ was added and incubated at 37 °C for 24 h. At 1, 3, and 7 days after Hase digestion, the viabilities of the cells were measured with live/dead staining (Invitrogen, USA). In detail, the constructs were rinsed with PBS and incubated with 2 μM of calcein-AM and 4 μM of ethidium homodimer-1 for 30 min and then observed by fluorescence microscopy. The numbers of live cells and dead cells were quantified with ImageJ. Additionally, cellular metabolic activities were assessed using MTS assay with the CellTiter 96^®^ AQueous Assay. Moreover, the supernatants from cell cultures at 5, 10, and 15 days after Hase digestion were collected and stored at −80 °C. The quantifications of ALB and urea secretions were conducted using an enzyme-linked immunosorbent assay (ELISA) kit for ALB (Abcam) and urea assay kit (Sigma-Aldrich), respectively, according to the manufacturers’ instructions.

The samples were collected at 7 and 14 days after Hase digestion for the evaluations of gene expressions. The samples were immersed in TRIzol (ThermoFisher) and homogenized using the Precellys lysing kits (Precellys, France) to isolate the total RNAs. Then, the first-strand cDNA was synthesized using the SuperScript^®^ VILO™ cDNA Synthesis Kit (Invitrogen) according to the instructions of the manufacturer. The quantitative reverse transcription-polymerase chain reaction (qRT-PCR) was conducted with the PowerTrack SYBR Green Master Mix (ThermoFisher). The primers were obtained from Integrated DNA Technologies (USA) and their information is listed in Supplementary Table 4. RT-PCR analysis was then run on the QuantStudio 5 Real-Time PCR instrument (ThermoFisher) and in QuantStudio Real-Time PCR Software (v1.5.1, Applied Biosystems, USA) with duplicate copies and the results were normalized against the housekeeping *GAPDH* gene. For immunostaining, the samples were fixed, permeabilized, and blocked by 4% paraformaldehyde, 0.03 v/v% Triton X-100, and 5% BSA, respectively. Subsequently, the samples were incubated with the primary antibodies against ALB (Abcam, dilution 1:500, cat no. ab207327), E-cadherin (Abcam, dilution 1:500, cat no. ab231303), CYP1A2 (Abcam, dilution 1:500, cat no. ab22717), or CYP3A4 (Abcam, dilution 1:250, cat no. MA3-032) overnight at 4 °C, and then incubated with the corresponding secondary antibody (Alexa Fluor^®^-488 goat anti-rabbit IgG, cat no. A32731; Alexa Fluor^®^-594 donkey anti-mouse IgG, cat no. A32744; Alexa Fluor^®^-594 donkey anti-mouse IgG, cat no. A32744; Alexa Fluor^®^-488 goat anti-mouse IgG, cat no. A32723; dilution 1:500) at 37 °C for 2 h, which was followed by DAPI counterstaining of the nuclei. The samples were then visualized using a confocal microscope, and the integrated optical densities (IODs) of fluorescence were analyzed with ImageJ.

### Casting hydrogel constructs encapsulating HepG2/C3A cells or C2C12 myoblasts

The 5% GelMA solutions containing HepG2/C3A cells (8 ×1 0^6^ cells mL^−1^), or the 7.5% GelMA solutions containing C2C12 myoblasts (6 × 10^6^ cells mL^−1^) were cast in rectangle PDMS molds (4 × 4 × 2 mm^3^) and subsequently crosslinked through exposure to ultraviolet (UV) irradiation (10 mW cm^−2^, 360–480 nm, 40 s, OmniCure S2000, Excelitas, USA). At 1, 3, and 7 days after culture, the viabilities of the HepG2/C3A cells were measured with live/dead staining and then observed by fluorescence microscopy. The numbers of live cells and dead cells were quantified with ImageJ. Moreover, cellular metabolic activities were assessed by MTS assay with the CellTiter 96^®^ AQueous Assay. The C2C12 myoblast samples were collected on day 3 after the casting and were stained with Alexa Fluor 594 Phalloidin (ThermoFisher, dilution 1:400, cat no. A12381) for F-actin observation. The images of C2C12 myoblasts under bright-field and fluorescence were then captured using a microscope.

### Brain tissue-bioprinting with SNaPs and characterizations

Human embryonic stem cells (hESCs) were cultured and maintained with mTeSR plus medium (Stem Cell Technologies, USA) on Geltrex (ThermoFisher)-coated tissue culture dishes. H01 hESCs were maintained in 5% CO_2_ incubators at 37 °C and passaged every 4–5 days as small aggregates after accutase (Innovative Cell Technologies, USA)-treatment. After dissociation, 10-μM ROCK-inhibitor (Sigma-Aldrich) was added to the cell culture for 24 h to prevent cell death. For NGN2 viral transduction, TetO-Ngn2-Puromycin and Ubq-rtTA plasmid constructs were obtained from the Wernig Lab and packaged as high-titer lentiviruses (Alstem, USA). The cells were dissociated and resuspended in virus-containing mTeSR medium supplemented with ROCK-inhibitor at a multiplicity of infection (MOI) of 1 to 3. Transduced cells were maintained in mTeSR for up to ten passages, with a 70–90% transduction efficiency.

The protocol for inducing SNaPs was previously described^56^. In brief, the cells were maintained in mTeSR medium and were fed with induction medium containing DMEM/F12 (ThermoFisher) supplemented with 20% glucose (1.5 v/v%, ThermoFisher), 1:100 glutamax (ThermoFisher), 1:100 N2 supplement (ThermoFisher), 2 µg mL^−1^ of doxycycline (Sigma-Aldrich), 10 µM of SB431542 (Tocris, UK), 200 nM of LDN-193189 (Stemgent, USA), and 2 µM of XAV939 (Stemgent) on day 1. After 24-h induction, medium was changed to selection medium with DMEM/F12, 20% glucose (1.5 v/v%), 1:100 glutamax (ThermoFisher), 1:100 N2 supplement, 2-µg mL^−1^ doxycycline, 5-µg mL^−1^ puromycin (ThermoFisher), 100-nM LDN-193189, 5-µM SB431542, and 1-µM XAV939 for 24 h. On day 3, SNaPs were dissociated and replated at a density of 1.2 × 10^5^ cells cm^−2^ and maintained in SNaP maintenance medium using DMEM/F12 supplemented with 1:100 glutamax, 1:100 N2 supplement, 1:100 MEM-NEAA (ThermoFisher), 1:50 B27 minus vitamin A (ThermoFisher), 10-ng mL^−1^ recombinant human basic fibroblast growth factor (bFGF, ThermoFisher), and 10-ng mL^−1^ recombinant human epidermal growth factor (EGF, R&D Systems, USA). On the same day, 5-µg mL^−1^ puromycin and 10-µM Y-27632 (Tocris) were added to the medium. About 12–24 h after replating, SNaPs were fed with a maintenance medium and passaged every 5–7 days for future experiments. For spontaneous differentiation, SNaPs were differentiated in base differentiation medium containing DMEM/F12 supplemented with 1:50 glutamax, 1:100 MEM-NEAA, 1:50 B27, 1:100 N2 supplement, and 10% FBS. 2-µg mL^−1^ doxycycline was added to the base differentiation medium for 2–3 days to direct neuronal differentiation. The medium was then exchanged to remove doxycycline, and cells were fed 2–3 times a week using base differentiation medium supplemented with 10-ng mL^−1^ brain-derived neurotrophic factor (BDNF, ThermoFisher), 10-ng mL^−1^ ciliary neurotrophic factor (CDNF, ThermoFisher), and 10-ng mL^−1^ glial cell line-derived neurotrophic factor (CNTF, ThermoFisher).

For the bioprinting of SNaPs, the cells (1 × 10^7^ cells mL^−1^) were mixed with GelMA/HAMA (2.5%/1.5%) and 1-mM/10-mM Ru/SPS as the bioink. The bioprinting was conducted with a 30-s exposure time for each layer (400 µm of layer thickness) to achieve the final construct in 150 s. The bioprinted constructs (cubes of 4 × 4 × 2 mm^3^) were rinsed with PBS three times before adding Hase (500 or 1000 U mL^−1^) for enzymatic digestion in the maintenance medium for culture. By contrast, the bioink of 10% GelMA (minimally printable concentration of GelMA for DLP in the absence of HAMA), which has been widely used in 3D bioprinting of brain-mimics (though again, at this concentration the cell functions would be very limited as also shown in literature^20^) containing 1-mM/10-mM Ru/SPS was adopted as the control group. The Hase was removed at 24 h after digestion, and the bioprinted SNaPs were cultured in the maintenance medium for 3 extra days with medium changed every day.

The bioprinted cells were stained for Ki67 as a proliferation marker^83^. The samples were fixed in 4% paraformaldehyde for 15 min, permeabilized with 0.03 v/v% of Triton X-100, and then blocked with 5% BSA for 1 h at room temperature. After being incubated with Ki67 antibody conjugated to Alexa Fluor^®^ 594 (Abcam, dilution 1:100, cat no. ab216709) overnight at 4 °C, or nestin antibody (Stem cell technologies, dilution 1:1000, cat no. 60091) overnight at 4 °C and secondary antibody (Alexa Fluor^®^-594 goat anti-mouse IgG, dilution 1:500, cat no. A32744) at 37 °C for 2 h, the stained samples were observed with the confocal microscope. The number of Ki67^+^ cells was quantified by ImageJ. Moreover, live/dead staining was carried out on days 1, 2, and 3 after digestion. The SNaP differentiation was induced at day 3 after Hase digestion by adding the differentiation medium, which was changed every 3 days during culture. To evaluate the differentiation of bioprinted SNaPs, the samples were fixed, permeabilized, and blocked with 4% paraformaldehyde, 0.03 v/v% Triton X-100, and 5% BSA, respectively. The samples were incubated with the primary antibodies of GFAP (Abcam, dilution 1:100, cat no. AB5804), TUJ1 (Abcam, dilution 1:1000, cat no. NL1195V), synaptophysin (Sigma-Aldrich, dilution 1:100, cat no. ZRB1365), or S100B (Sigma-Aldrich, dilution 1:1000, cat no. S2532) overnight at 4 °C, and then incubated with the corresponding secondary antibody (Alexa Fluor^®^-647 donkey anti-rabbit, cat no. A-31573; Alexa Fluor^®^-488 goat anti-mouse IgG, cat no. A32744; Alexa Fluor^®^-488 goat anti-rabbit IgG, cat no. A32731; or Alexa Fluor^®^-488 goat anti-mouse IgG, cat no. A32723; dilution 1:500) at 37 °C for 2 h, which was followed by DAPI staining for the nuclei. The samples were then visualized using confocal microscopy.

MEA recording of 3D-bioprinted mini-brains was performed from week 1 to week 4 after differentiation (day 10 to day 31 after digestion). The samples containing SNaPs were plated on Geltrex-coated 12-well MEA plates (Axion Biosystems, USA) in the differentiation medium. The samples were fed 2–3 times a week with partial medium change. Neuronal activities were measured every week using the Maestro 12-well MEA plate system (Axion Biosystems) for 5 min. After 4 weeks of culture in MEA plate, synaptic contents were tested using pharmacological inhibitors of neurotransmitter receptors. Specifically, neuronal activity was measured for 5 min by adding DMSO control before adding 10 µM of NBQX (Abcam) or 10 µM of D-APV (Abcam). The samples were incubated for 5–10 min, and neuronal activities were assessed again for 5 min. All the MEA data were analyzed using the Axion Integrated Studio 2.4.2 and the Neural Metric Tool (Axion Biosystems).

### RNA-seq and data analyses

Using TRIzol and Precellys lysing kits, RNA was extracted from 3D-bioprinted mini-brain samples, which were cultured for 28 days after differentiation. RNA-seq was processed and analyzed by Beijing Genomics Institute (BGI, China). Library quality and quantitation were conducted using a Qubit fluorometer (ThermoFisher) and an Agilent 2100 Bioanalyzer (Agilent Technologies, USA). After quality control, the constructed libraries were amplified with phi29 to make DNA nanoball (DNB), which had >300 copies of one molecular. The DNBs were loaded into the patterned nanoarray and single-end 50 (pair-end 100/150) bases reads were generated in the way of combinatorial Probe Anchor Synthesis (cPAS). We applied Bowtie2 for mapping the clean reads to the reference gene sequence, and then used RSEM to calculate the gene expression level of each sample. Significant DEGs were determined by false discovery rate (FDR) < 0.05. According to the results of differential gene detection, the R package heatmap was used to perform hierarchical clustering analysis on the union set differential genes. PCA was performed for comparison between the samples (GM and Hase-1000). For GO and KEGG pathway enrichment analyses, all DEGs were mapped to terms in the KEGG and GO databases and queried for significantly enriched terms. Pathway with *Q*-value (corrected *P*-value) < 0.05 was defined as the pathway that is significantly enriched in differentially expressed genes. GSEA was performed with the database of GSEA MSigDB C5 (GO) biological processes.

### Statistical analyses

The data are presented as means ± standard deviations (SDs). All statistical analyses were performed with one-way ANOVA followed by a Tukey’s Honest Significant Difference test or a two-tailed student’s *t*-test. *p* < 0.05 was considered statistically significant. GraphPad Prism software (v8.2.0) was used for all statistical analyses, and Microsoft excel (v16.54) was used for data handling

### Reporting summary

Further information on research design is available in the Nature Research Reporting Summary linked to this article.

## Supplementary information

Supplementary Information

Reporting Summary

## Data Availability

The raw RNA-Seq data generated in this study have been deposited in the National Center for Biotechnology Information (NCBI) Sequence Read Archive (SRA) as a BioProject under the Accession Number “PRJNA836719”. All other relevant data supporting the key findings of this study are available within the article and its Supplementary Information files as well as Source Data. Requests for additional raw images and materials will be promptly reviewed by the Brigham and Women’s Hospital, and will be released via a Material Transfer Agreement. Source data are provided with this paper.

## Source data

Source Data

## Code availability

The codes developed in this study are available from the corresponding author upon reasonable request. All requests will be promptly reviewed by the Brigham and Women’s Hospital to verify whether the request is subject to any intellectual property or confidentiality obligations. Any codes that can be shared will be released *via* a Material Transfer Agreement.

## References

[1] Mota C, Camarero-Espinosa S, Baker MB, Wieringa P, Moroni L (2020). Bioprinting: from tissue and organ development to in vitro models. Chem. Rev..

[2] Moroni L (2018). Biofabrication strategies for 3D in vitro models and regenerative medicine. Nat. Rev. Mater..

[3] Murphy SV, Atala A (2014). 3D bioprinting of tissues and organs. Nat. Biotechnol..

[4] Murphy S. V., De Coppi P. & Atala A. Opportunities and challenges of translational 3D bioprinting. *Nat. Biomed. Eng.* **4**, 370–380 (2019). 10.1038/s41551-019-0471-7 31695178

[5] Heinrich MA (2019). 3D Bioprinting: from benches to translational applications. Small.

[6] Jorgensen AM, Yoo JJ, Atala A (2020). Solid organ bioprinting: strategies to achieve organ function. Chem. Rev..

[7] Levato R (2020). From shape to function: the next step in bioprinting. Adv. Mater..

[8] Lim KS (2020). Fundamentals and applications of photo-cross-linking in bioprinting. Chem. Rev..

[9] Yu C (2020). Photopolymerizable biomaterials and light-based 3D printing strategies for biomedical applications. Chem. Rev..

[10] Lee M, Rizzo R, Surman F, Zenobi-Wong M (2020). Guiding lights: tissue bioprinting using photoactivated materials. Chem. Rev..

[11] Grigoryan B (2019). Multivascular networks and functional intravascular topologies within biocompatible hydrogels. Science.

[12] West-Livingston LN, Park J, Lee SJ, Atala A, Yoo JJ (2020). The role of the microenvironment in controlling the fate of bioprinted stem cells. Chem. Rev..

[13] Li W (2020). Recent advances in formulating and processing biomaterial inks for vat polymerization-based 3D printing. Adv. Healthc. Mater..

[14] Matai I, Kaur G, Seyedsalehi A, McClinton A, Laurencin CT (2020). Progress in 3D bioprinting technology for tissue/organ regenerative engineering. Biomaterials.

[15] Schwab A (2020). Printability and shape fidelity of bioinks in 3D bioprinting. Chem. Rev..

[16] Liu W (2017). Extrusion bioprinting of shear-thinning gelatin methacryloyl bioinks. Adv. Healthc. Mater..

[17] Ying G-L (2018). Aqueous two-phase emulsion bioink-enabled 3D bioprinting of porous hydrogels. Adv. Mater..

[18] Ouyang L (2020). Expanding and optimizing 3D bioprinting capabilities using complementary network bioinks. Sci. Adv..

[19] Ma X (2016). Deterministically patterned biomimetic human iPSC-derived hepatic model via rapid 3D bioprinting. Proct Natl Acad. Sci. USA.

[20] Tang M (2020). Three-dimensional bioprinted glioblastoma microenvironments model cellular dependencies and immune interactions. Cell Res.

[21] Sun, Y. et al. Modeling the printability of photocuring and strength adjustable hydrogel bioink during projection-based 3D bioprinting. *Biofabrication*10.1088/1758-5090/aba413 (2021). 10.1088/1758-5090/aba413 32640425

[22] Beh CW (2021). A fluid-supported 3D hydrogel bioprinting method. Biomaterials.

[23] Axpe E, Orive G, Franze K, Appel EA (2020). Towards brain-tissue-like biomaterials. Nat. Commun..

[24] Ying G, Jiang N, Yu C, Zhang YS (2018). Three-dimensional bioprinting of gelatin methacryloyl (GelMA). Bio-Des. Manuf..

[25] Nie L., Wang C., Deng Y. & Shavandi A. Bio-inspired hydrogels via 3D bioprinting. *Biomimetics*. 10.5772/intechopen.94985 (2020).

[26] Dhand AP, Galarraga JH, Burdick JA (2021). Enhancing biopolymer hydrogel functionality through interpenetrating networks. Trends Biotechnol..

[27] Highley CB, Rodell CB, Burdick JA (2015). Direct 3D printing of shear-thinning hydrogels into self-healing hydrogels. Adv. Mater..

[28] Hinton TJ (2015). Three-dimensional printing of complex biological structures by freeform reversible embedding of suspended hydrogels. Sci. Adv..

[29] Bhattacharjee T (2015). Writing in the granular gel medium. Sci. Adv..

[30] Zhou F (2020). Rapid printing of bio-inspired 3D tissue constructs for skin regeneration. Biomaterials.

[31] Kelly BE (2019). Volumetric additive manufacturing via tomographic reconstruction. Science.

[32] Bernal PN (2019). Volumetric bioprinting of complex living‐tissue constructs within seconds. Adv. Mater..

[33] Tumbleston JR (2015). Continuous liquid interface production of 3D objects. Science.

[34] Walker David A, Hedrick James L, Mirkin Chad A (2019). Rapid, large-volume, thermally controlled 3D printing using a mobile liquid interface. Science.

[35] Anandakrishnan N (2021). Fast stereolithography printing of large-scale biocompatible hydrogel models. Adv. Healthc. Mater..

[36] Lim KS (2018). Bio-resin for high resolution lithography-based biofabrication of complex cell-laden constructs. Biofabrication.

[37] Huh J (2021). Combinations of photoinitiator and UV absorber for cell-based digital light processing (DLP) bioprinting. Biofabrication.

[38] Sayar S, Özdemir Y (1998). First-derivative spectrophotometric determination of ponceau 4R, sunset yellow and tartrazine in confectionery products. Food Chem..

[39] Wang M (2022). Digital light processing based bioprinting with composable gradients. Adv. Mater..

[40] Li W (2021). A smartphone‐enabled portable digital light processing 3D printer. Adv. Mater..

[41] Ma C. et al. Photoacoustic imaging of 3D-printed vascular networks. *Biofabrication* **14**, 025001 (2022). 10.1088/1758-5090/ac49d5 PMC8885332 35008080

[42] Levato R (2021). High-resolution lithographic biofabrication of hydrogels with complex microchannels from low-temperature-soluble gelatin bioresins. Mater. Today Bio.

[43] Kadokawa J (2011). Precision polysaccharide synthesis catalyzed by enzymes. Chem. Rev..

[44] Guimarães CF, Gasperini L, Marques AP, Reis RL (2020). The stiffness of living tissues and its implications for tissue engineering. Nat. Rev. Mater..

[45] Greene T., Lin T. Y., Andrisani O. M., Lin C. C. Comparative study of visible light polymerized gelatin hydrogels for 3D culture of hepatic progenitor cells. *J. Appl. Polym. Sci.*10.1002/app.44585 (2017).

[46] Lee EJ (2018). Establishment of stably expandable induced myogenic stem cells by four transcription factors. Cell Death Dis..

[47] Le Grand F, Rudnicki MA (2007). Skeletal muscle satellite cells and adult myogenesis. Curr. Opin. Cell Biol..

[48] Jensen JH (2020). Large-scale spontaneous self-organization and maturation of skeletal muscle tissues on ultra-compliant gelatin hydrogel substrates. Sci. Rep..

[49] Maharjan S (2021). 3D human nonalcoholic hepatic steatosis and fibrosis models. Bio-Des. Manuf..

[50] Billiet T, Gevaert E, De Schryver T, Cornelissen M, Dubruel P (2014). The 3D printing of gelatin methacrylamide cell-laden tissue-engineered constructs with high cell viability. Biomaterials.

[51] Ma X (2018). Rapid 3D bioprinting of decellularized extracellular matrix with regionally varied mechanical properties and biomimetic microarchitecture. Biomaterials.

[52] Lewis PL, Green RM, Shah RN (2018). 3D-printed gelatin scaffolds of differing pore geometry modulate hepatocyte function and gene expression. Acta Biomater..

[53] Lacour SP, Courtine G, Guck J (2016). Materials and technologies for soft implantable neuroprostheses. Nat. Rev. Mater..

[54] Liu Z, Tang M, Zhao J, Chai R, Kang J (2018). Looking into the future: toward advanced 3D biomaterials for stem‐Cell‐based regenerative medicine. Adv. Mater..

[55] Mertens J, Marchetto MC, Bardy C, Gage FH (2016). Evaluating cell reprogramming, differentiation and conversion technologies in neuroscience. Nat. Rev. Neurosci..

[56] Wells M. F., et al. Genome-wide screens in accelerated human stem cell-derived neural progenitor cells identify Zika virus host factors and drivers of proliferation. *bioRxiv* **116**, 9527–9532 (2018).

[57] Lee HJ (2019). Non-proteolytic ubiquitination of Hexokinase 2 by HectH9 controls tumor metabolism and cancer stem cell expansion. Nat. Commun..

[58] Peifer C, Alessi DR (2008). Small-molecule inhibitors of PDK1. ChemMedChem.

[59] Jeon Y (2021). In vivo gene delivery of STC2 promotes axon regeneration in sciatic nerves. Mol. Neurobiol..

[60] Zhou S, Zhou J (2014). Neuritin, a neurotrophic factor in nervous system physiology. Curr. Med Chem..

[61] Stogsdill JA (2017). Astrocytic neuroligins control astrocyte morphogenesis and synaptogenesis. Nature.

[62] Carletti B, Piemonte F, Rossi F (2011). Neuroprotection: the emerging concept of restorative neural stem cell biology for the treatment of neurodegenerative diseases. Curr. Neuropharmacol..

[63] Mo JS, Park HW, Guan KL (2014). The Hippo signaling pathway in stem cell biology and cancer. EMBO Rep..

[64] Ingber DE (2006). Cellular mechanotransduction: putting all the pieces together again. FASEB J..

[65] Orr AW, Helmke BP, Blackman BR, Schwartz MA (2006). Mechanisms of mechanotransduction. Developmental cell.

[66] Goldman S (2005). Stem and progenitor cell-based therapy of the human central nervous system. Nat. Biotechnol..

[67] Freeman MR (2010). Specification and morphogenesis of astrocytes. Science.

[68] Romero-Ramos M (2002). Neuronal differentiation of stem cells isolated from adult muscle. J. Neurosci. Res.

[69] Steiner J (2007). Evidence for a wide extra-astrocytic distribution of S100B in human brain. BMC Neurosci..

[70] Salto C (2008). Control of neural stem cell adhesion and density by an electronic polymer surface switch. Langmuir.

[71] Uto K, Tsui JH, DeForest CA, Kim DH (2017). Dynamically tunable cell culture platforms for tissue engineering and mechanobiology. Prog. Polym. Sci..

[72] Greve F (2007). Molecular design and characterization of the neuron-microelectrode array interface. Biomaterials.

[73] Bakkum DJ (2013). Tracking axonal action potential propagation on a high-density microelectrode array across hundreds of sites. Nat. Commun..

[74] Pitt D, Werner P, Raine CS (2000). Glutamate excitotoxicity in a model of multiple sclerosis. Nat. Med.

[75] Murphy GG, Glanzman DL (1999). Cellular analog of differential classical conditioning inAplysia: disruption by the NMDA receptor AntagonistDL-2-Amino-5-Phosphonovalerate. J. Neurosci..

[76] Yoon HJ (2016). Cold water fish gelatin methacryloyl hydrogel for tissue engineering application. PLoS One.

[77] Maharjan S (2021). Symbiotic photosynthetic oxygenation within 3D-bioprinted vascularized tissues. Matter.

[78] Zhu M (2019). Gelatin methacryloyl and its hydrogels with an exceptional degree of controllability and batch-to-batch consistency. Sci. Rep..

[79] Gong J (2020). Complexation-induced resolution enhancement of 3D-printed hydrogel constructs. Nat. Commun..

[80] Miri AK (2018). Microfluidics-enabled multimaterial maskless stereolithographic bioprinting. Adv. Mater..

[81] Hettiaratchi MH (2018). A rapid method for determining protein diffusion through hydrogels for regenerative medicine applications. APL Bioeng..

[82] Ferrenberg S, Reed SC, Belnap J (2015). Climate change and physical disturbance cause similar community shifts in biological soil crusts. Proc. Natl Acad. Sci. USA.

[83] Urruticoechea A, Smith IE, Dowsett M (2005). Proliferation marker Ki-67 in early breast cancer. J. Clin. Oncol..

