# Supplementary material for: Molecularly cleavable bioinks facilitate high-performance digital light processing-based bioprinting of functional volumetric soft tissues
Source: Nat Commun. 2022 Jun 9;13:3317. doi: 10.1038/s41467-022-31002-2 (PMC9184597; doi:10.1038/s41467-022-31002-2)
Supplement: Supplementary file 1 — Supplementary Information [file 41467_2022_31002_MOESM1_ESM.pdf]

Supplementary Information

Molecularly cleavable bioinks facilitate high-performance digital light processing-based bioprinting of functional volumetric soft tissues

Mian Wang[1, †], Wanlu Li[1, †], Jin Hao[2, 3, 4, †], Arthur Gonzales III[5], Zhibo Zhao[1], Regina Sanchez Flores[1], Xiao Kuang[1], Xuan Mu[1], Terry Ching[1, 6, 7, 8], Guosheng Tang[1], Zeyu Luo[1], Carlos Ezio Garciamendez Mijares[1], Jugal Kishore Sahoo[9], Michael F. Wells[2, 3, 4], Gengle Niu[2, 3], Prajwal Agrawal[1], Alfredo Quiñones-Hinojosa[10], Kevin Eggan[2, 3, 4], Yu Shrike Zhang[1, 3, *]

[1] Division of Engineering in Medicine, Department of Medicine, Brigham and Women's Hospital, Harvard Medical School, Cambridge, MA 02139, USA

[2] Department of Stem Cell and Regenerative Biology, Harvard University, Cambridge, MA, USA

[3] Harvard Stem Cell Institute, Harvard University, Cambridge, MA, USA

[4] Stanley Center for Psychiatric Research, Broad Institute of MIT and Harvard, Cambridge, MA, USA

[5] University of the Philippines Diliman, Quezon City, Metro Manila, Philippines

[6] Pillar of Engineering Product Development, Singapore University of Technology and Design, Singapore

[7] Digital Manufacturing and Design Centre, Singapore University of Technology and Design, Singapore

[8] Department of Biomedical Engineering, National University of Singapore, Singapore

[9] Department of Biomedical Engineering, Tufts University, Medford, MA, USA

[10] Departments of Neurosurgery, Oncology, Neuroscience, Mayo Clinic, Jacksonville, FL, USA

[†] Equal contribution

[*] Correspondences should be addressed to Y.S.Z. (email: yszhang@research.bwh.harvard.edu)

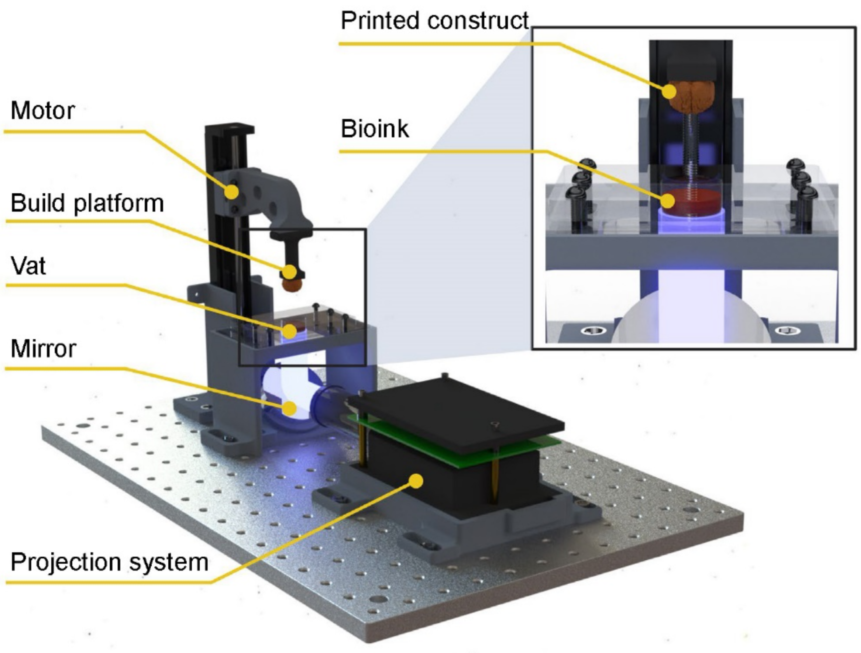

Supplementary Fig. 1 | Schematic of the in-house-built DLP-based 3D bioprinter.

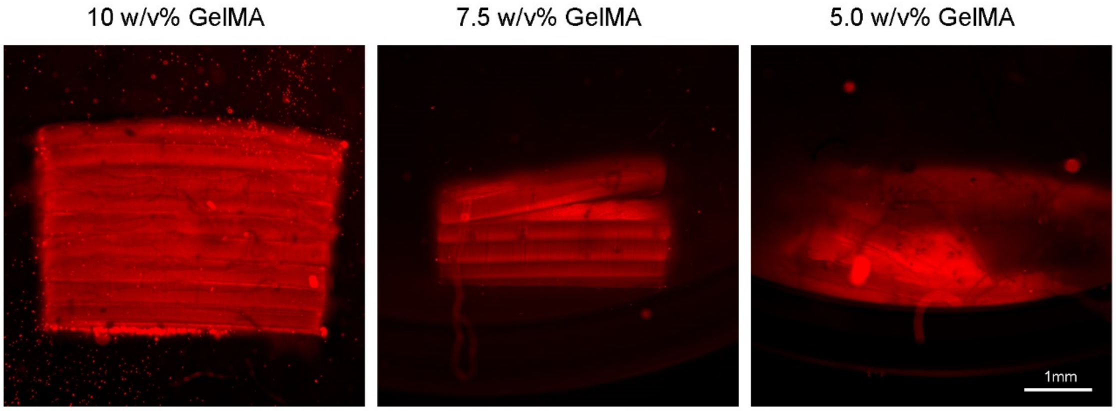

Supplementary Fig. 2 | Representative photographs showing printability of pure GelMA bioinks at different concentrations displaying good printability, partial printability, and non-printability. Images are representatives of $n = 3$ independent experiments.

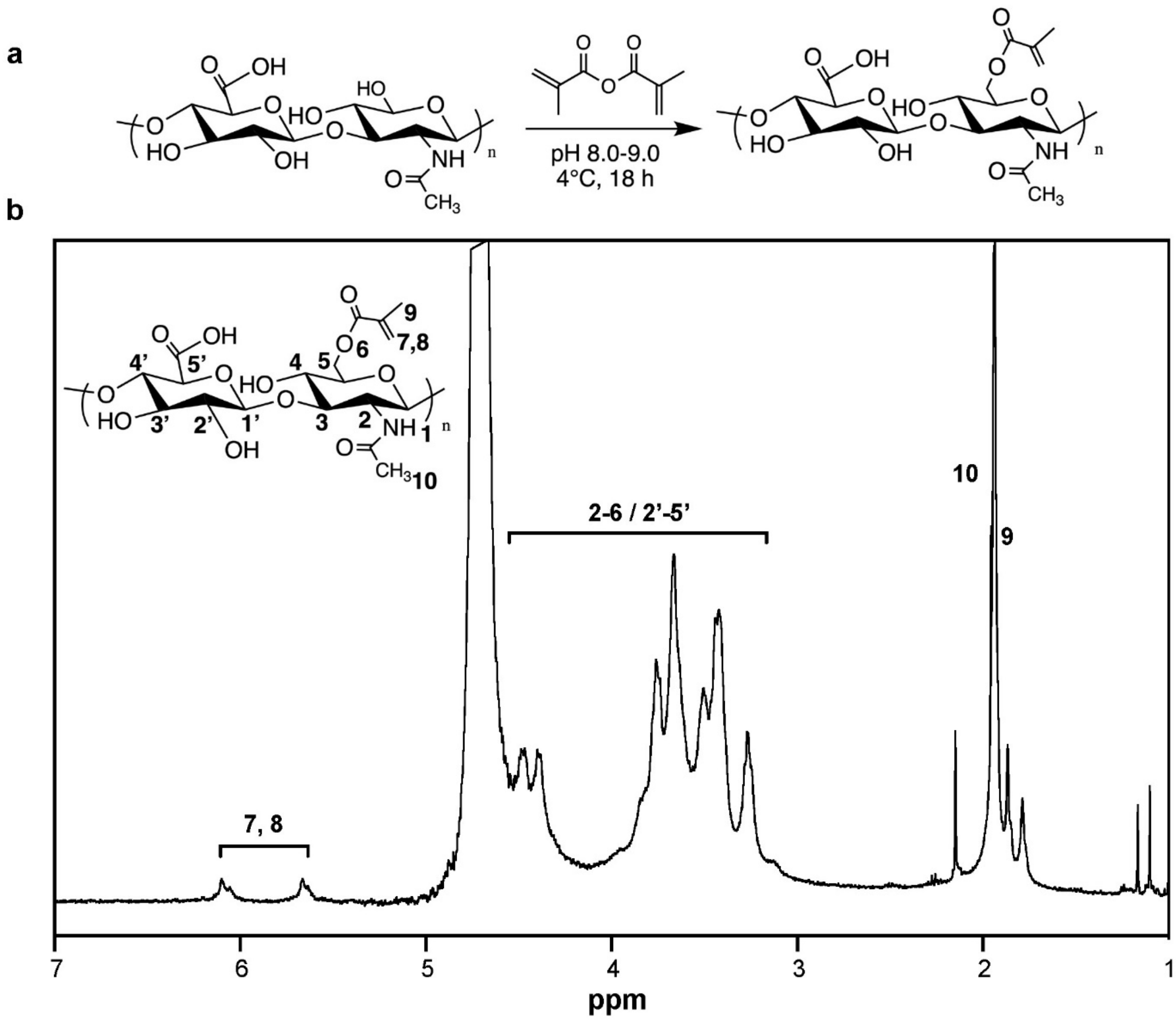

Supplementary Fig. 3 | Characterization of HAMA. **a**, Reaction schematic to prepare HAMA. **b**, Representative chemical structure and ^{1}H NMR spectrum of HAMA (M_w = 100 kDa).

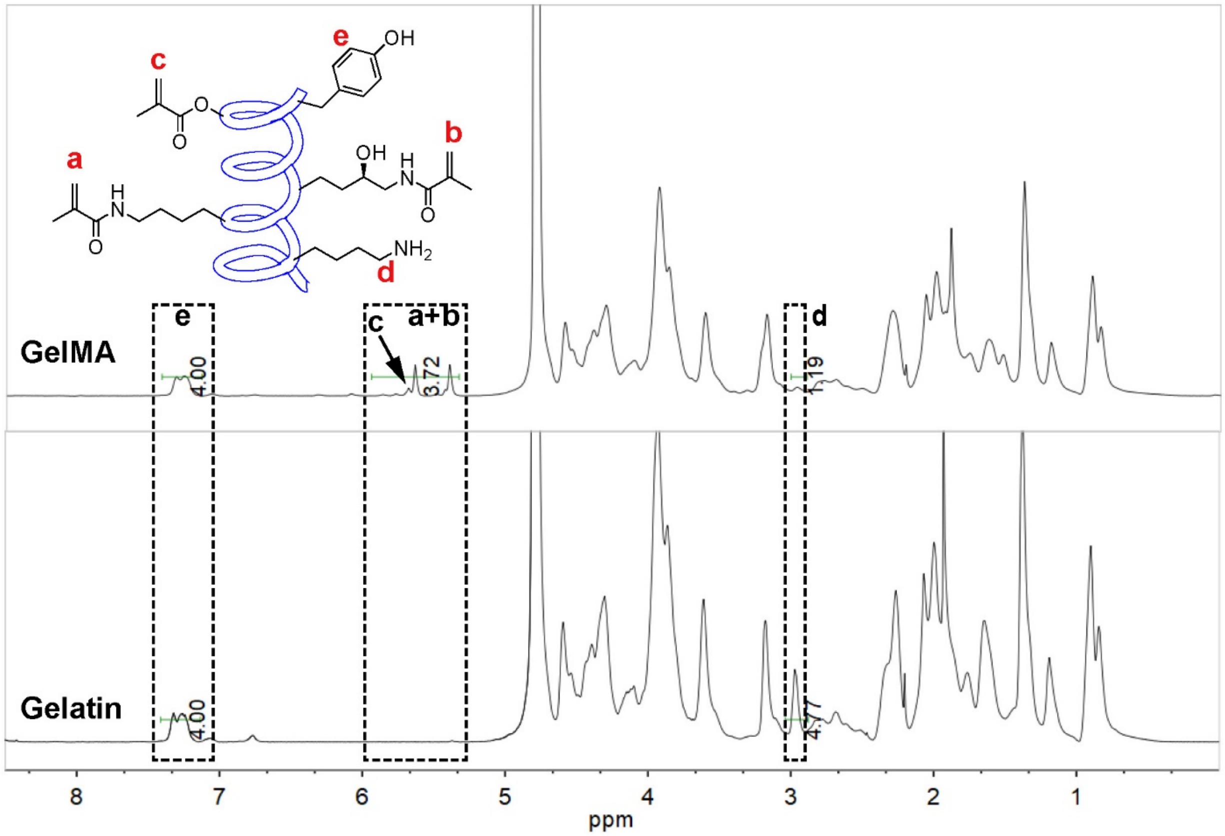

Supplementary Fig. 4 | ^{1}H-NMR spectra of GelMA and gelatin samples with marked peak assignments and areal integration.

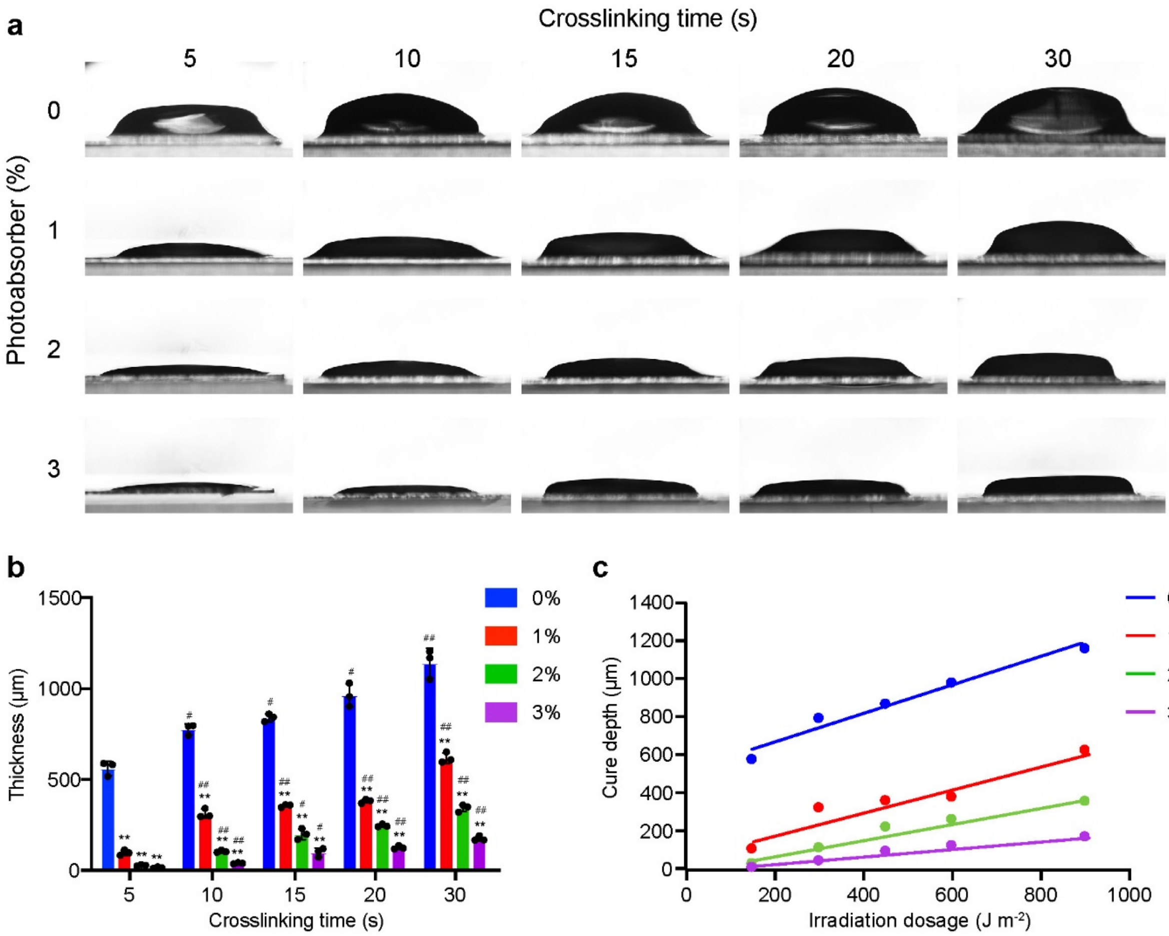

Supplementary Fig. 5 | Characterizations of working curves of the GelMA/HAMA inks (GelMA/HAMA, 5.0%/3.0%) under different crosslinking times. **a**, Optical images of the cross-sections of the printed constructs using the GelMA/HAMA (5.0%/3.0%) inks with different concentrations of photoabsorber (0, 1, 2, and 3%). **b**, Quantitative results of crosslinking thicknesses. $n = 3$; one-way ANOVA; $*p < 0.05$, $**p < 0.01$ (compared with the group of same exposure time but without photoabsorber); $^{\#}p < 0.05$, $^{\#\#}p < 0.01$ (compared with the corresponding results of same photoabsorber but at 5-s exposure time). Data are presented as mean values ± SDs. **c**, Working curves presenting photopolymerization kinetics of GelMA/HAMA (5.0%/3.0%) inks with different concentrations of photoabsorber and different exposure times. Source data are provided as a Source Data file.

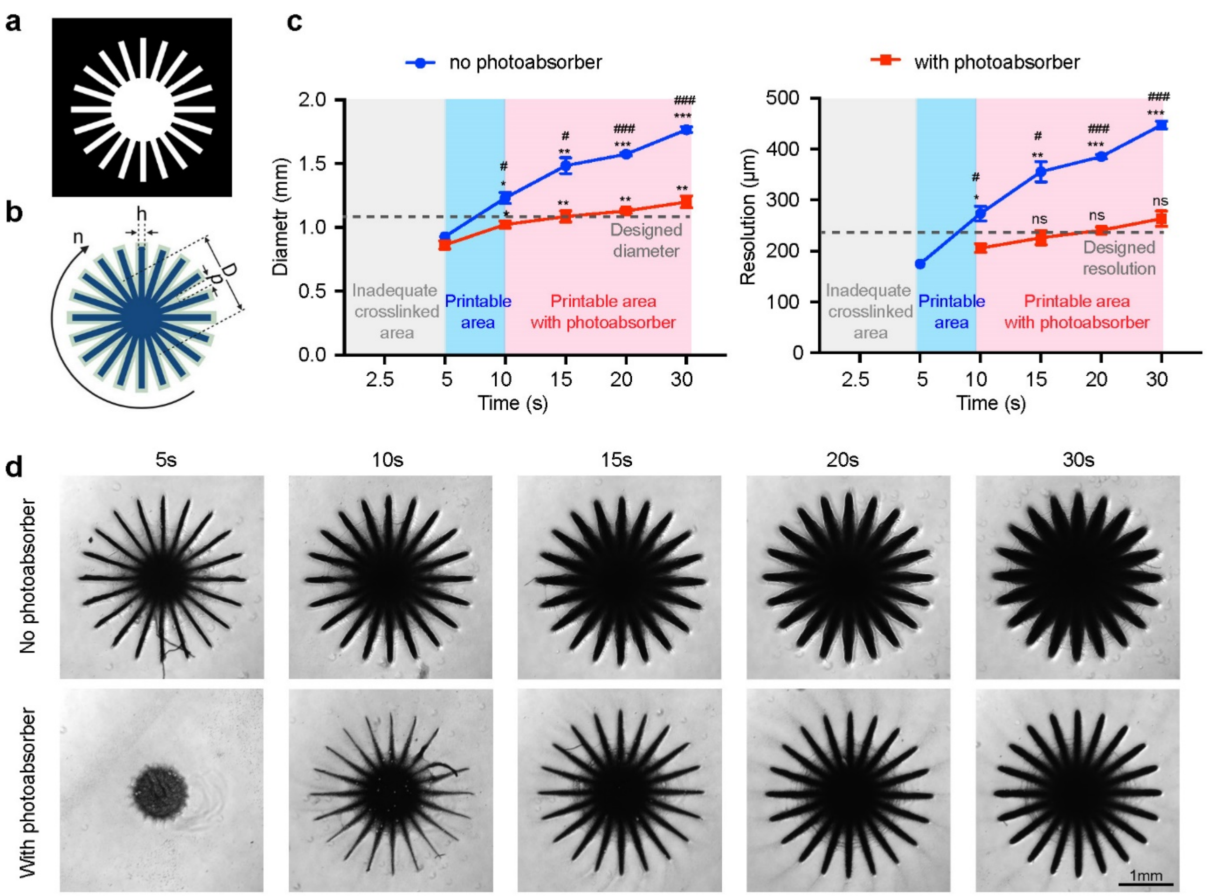

Supplementary Fig. 6 | Evaluations of printing resolutions with the GelMA/HAMA (5.0%/3.0%) inks. **a**, The digital radial pattern used for printing resolution test. **b**, Measurements of the designed model, where *p* is printing resolution, *D* is the diameter of a printed center radial pattern, and *h* is the width of the lines. **c**, Influence of exposure time on the printing resolution after adding 2.0% photoabsorber. $n = 3$; one-way ANOVA; $^{*}p < 0.05$, $^{**}p < 0.01$, $^{***}p < 0.001$ (compared with the group at 5-s or 10-s exposure time); $^{\#}p < 0.05$, $^{\#\#\#}p < 0.001$ (compared with the corresponding results of same exposure times but without photoabsorber). Data are presented as mean values ± SDs. **d**, Micrographs showing printed radial patterns in the absence and presence of photoabsorber (2.0%) under different exposure times. Source data are provided as a Source Data file.

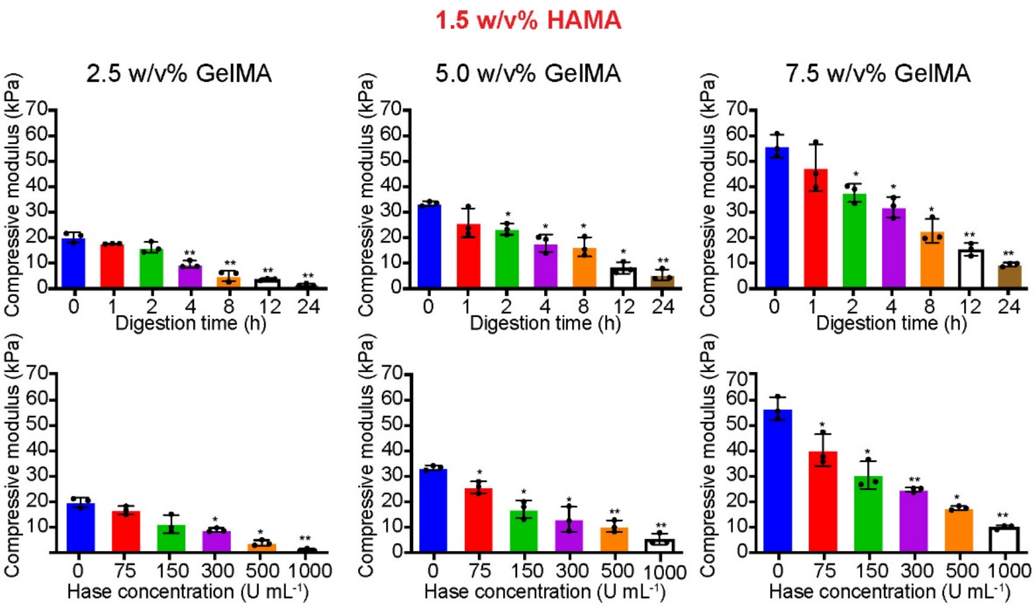

Supplementary Fig. 7 | Compressive moduli of hydrogel constructs made of GelMA/HAMA (1.5% of HAMA) inks at various GelMA concentrations (2.5%, 5.0%, and 7.5%), Hase digestion times (0, 1, 2, 4, 8, 12, and 24 h), and Hase concentrations (0, 75, 150, 300, 500, and 1,000 U mL^{-1}). $n = 3$; two-tailed student's t-test; *$p < 0.05$, **$p < 0.01$ (compared with the group without digestion, where the digestion time was 0 h or Hase concentration was 0 U mL^{-1}). Data are presented as mean values ± SDs. Source data are provided as a Source Data file.

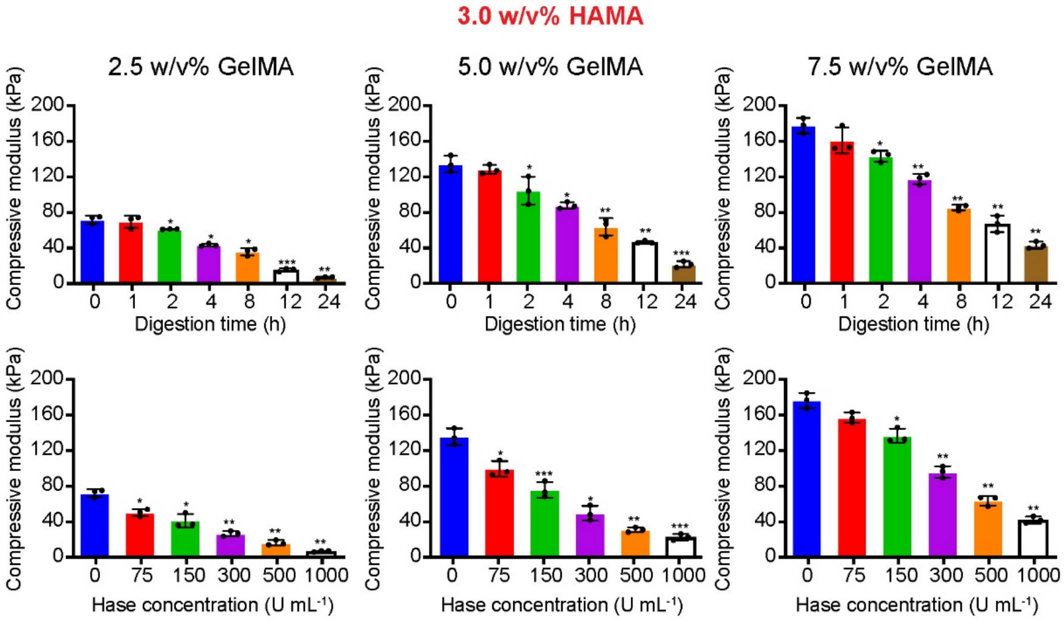

Supplementary Fig. 8 | Compressive moduli of hydrogel constructs made of GelMA/HAMA (3.0% of HAMA) inks at various GelMA concentrations (2.5%, 5.0%, and 7.5%), Hase digestion times (0, 1, 2, 4, 8, 12, and 24 h), and Hase concentrations (0, 75, 150, 300, 500, and 1,000 U mL^{-1}). $n = 3$; two-tailed student's t-test; *$p < 0.05$, **$p < 0.01$ (compared with the group without digestion, where the digestion time was 0 h or Hase concentration was 0 U mL^{-1}). Data are presented as mean values ± SDs. Source data are provided as a Source Data file.

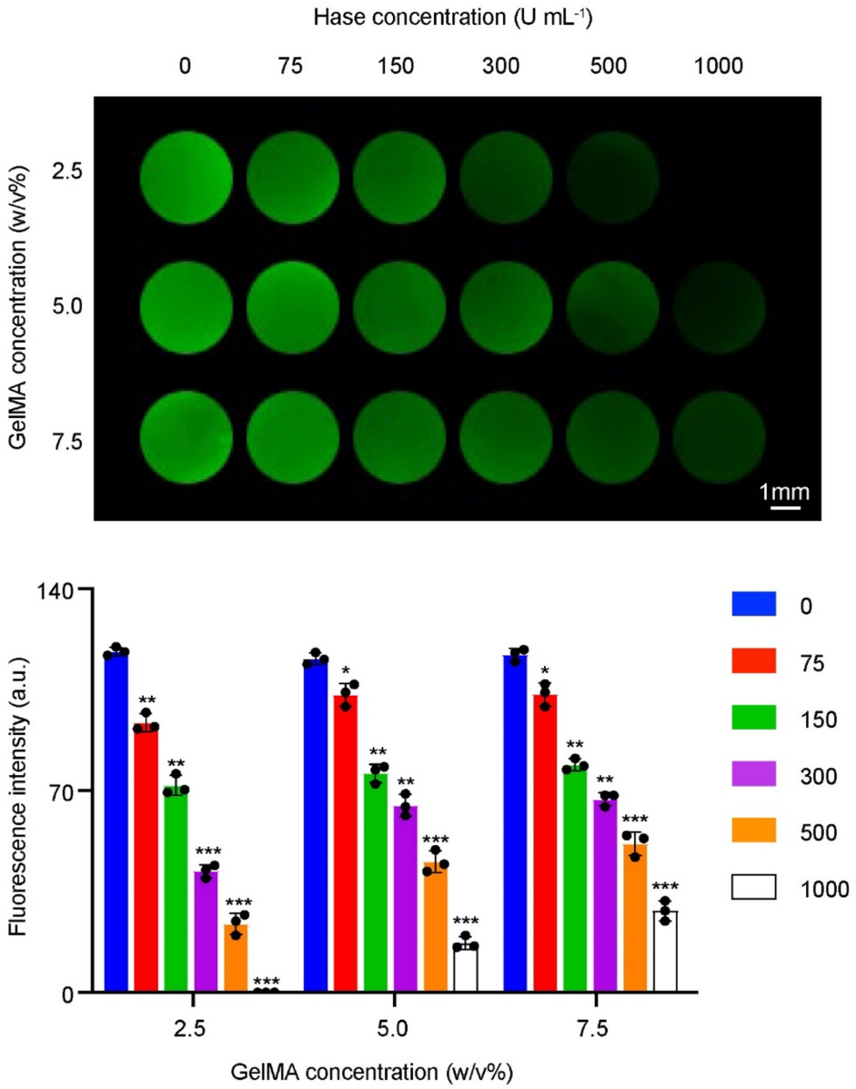

Supplementary Fig. 9 | Fluorescence microscopy images showing the 3D-printed hydrogels made of GelMA/HAMA-FITC (2.5%/1.5%, 5%/1.5%, and 7.5%/1.5%) to visualize the digestion processes of the HA component under different concentrations of Hase (0, 75, 150, 300, 500, and 1,000 U mL^{-1}) after 24 h of treatment. The bottom panel is the corresponding quantitative results of fluorescence intensities of the samples. $n = 3$; one-way ANOVA; $*p < 0.05$, $**p < 0.01$, $***p < 0.001$ (compared with the group without digestion where Hase concentration was 0 U mL^{-1}). Data are presented as mean values ± SDs. a.u., absolute unit. Source data are provided as a Source Data file.

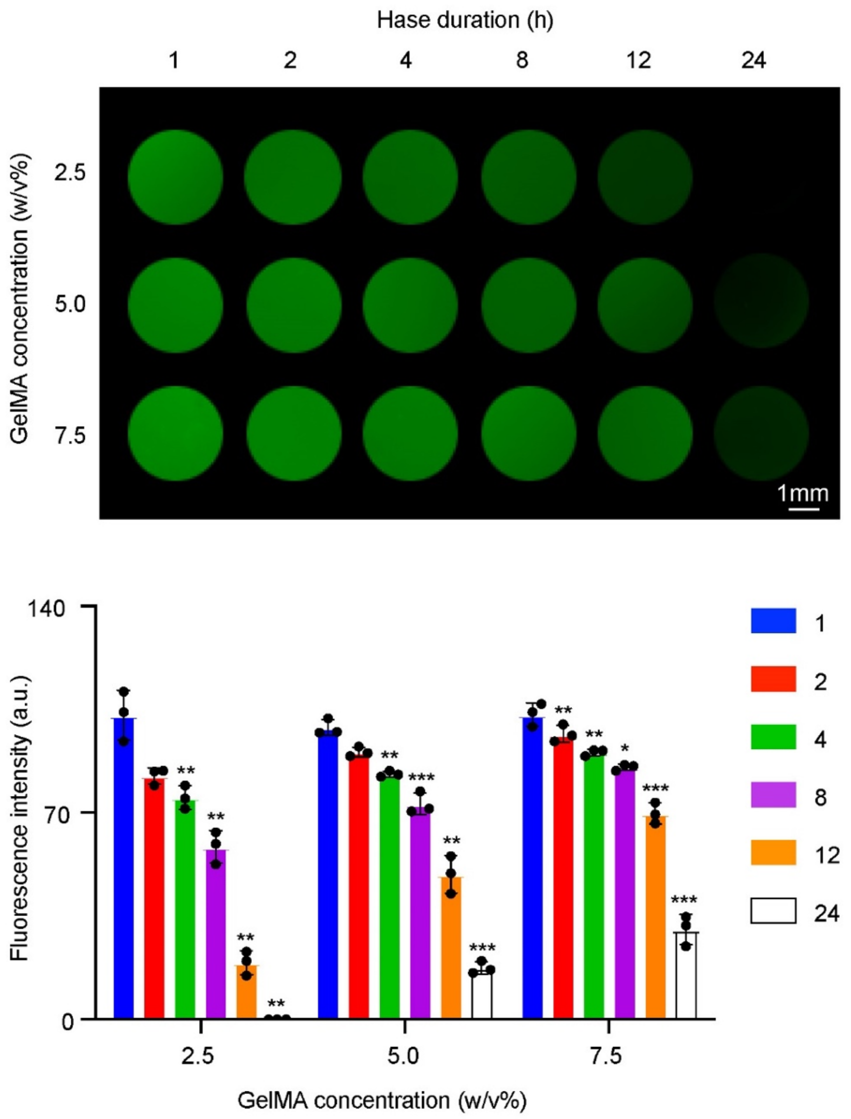

Supplementary Fig. 10 | Fluorescence microscopy images showing the 3D-printed hydrogels made of GelMA/HAMA-FITC (2.5%/1.5%, 5%/1.5%, and 7.5%/1.5%) to visualize the digestion processes of the HA component over different digestion times (0, 1, 2, 4, 8, 12, and 24 h) using 1,000 U mL^{-1} of Hase. The bottom panel is the corresponding quantitative results of fluorescence intensities of the samples. $n = 3$; one-way ANOVA; *$p < 0.05$, **$p < 0.01$, ***$p < 0.001$ (compared with the group without digestion where the digestion time was 0 h). Data are presented as mean values ± SDs. a.u., absolute unit. Source data are provided as a Source Data file.

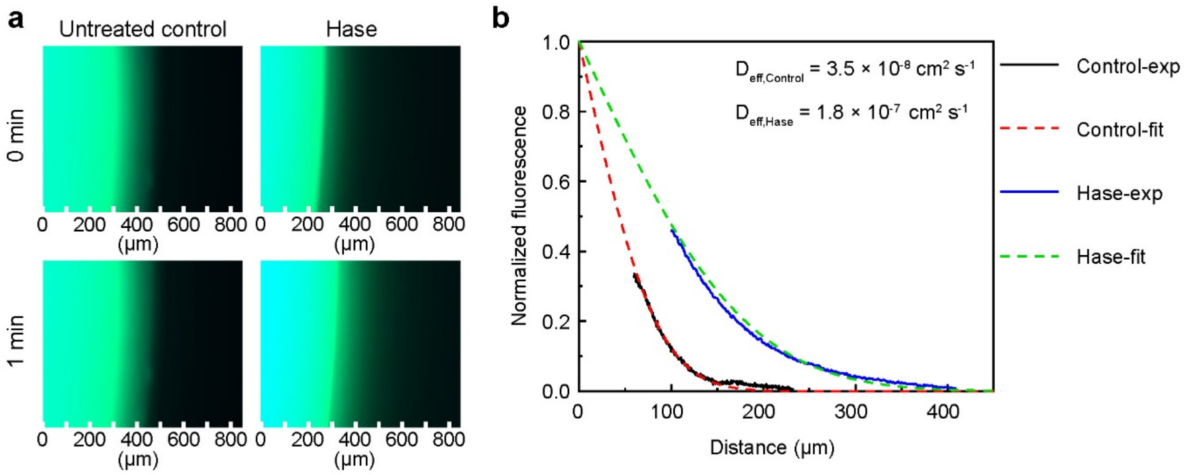

Supplementary Fig. 11 | **a**, Fluorescence microscopy images of GelMA/HAMA (5.0%/3.0%) hydrogels without (left) and with (right) Hase digestion (1,000 U mL^{-1}, 24 h, M_w = 55 kDa) after contacting with FITC-dextran (M_w = 60 kDa) for 1 min. **b**, Experimental and fitting results of normalized fluorescence intensities as a function of the distance from the FITC-dextran solution.

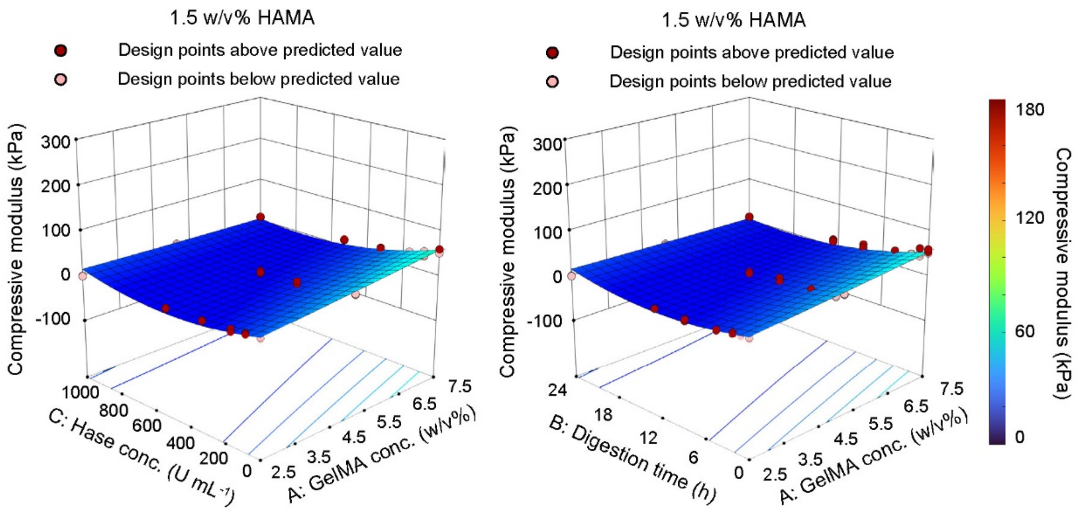

Supplementary Fig. 12 | 3D surface plots showing the effects of different parameters on the mechanical properties of the printed and digested hydrogel constructs containing 1.5% HAMA and different concentrations of GelMA.

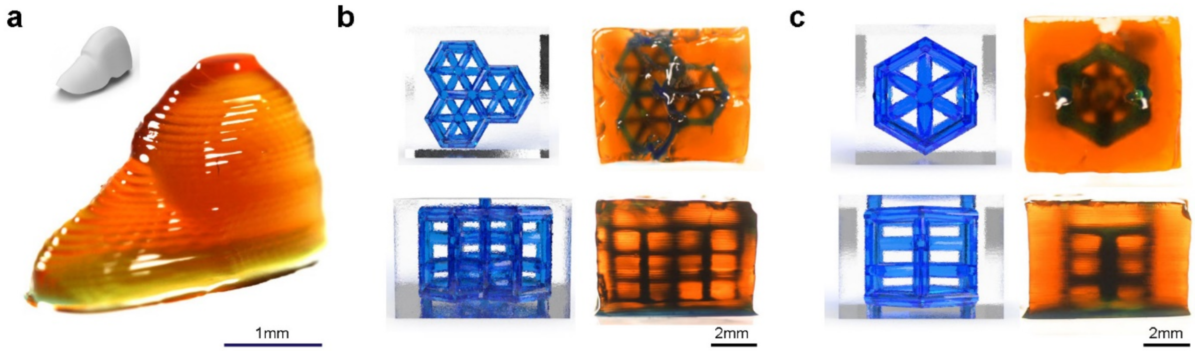

Supplementary Fig. 13 | Various printed volumetric hepatic models using the GelMA/HAMA (5.0%/3.0%) ink featuring complexity in multiple dimensions. **a**, Side view of the printed construct in a shape of the whole liver. **b**, Top view and side view of a cube containing three interconnected hepatic lobules with sinusoids. **c**, Top view and side view of a cube containing one unit of the hepatic lobule and associated sinusoids. The color dye could be perfused into the open channels showing the interconnectivity across the entire volume.

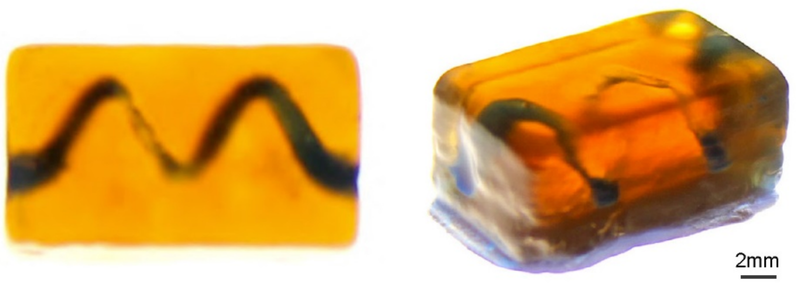

Supplementary Fig. 14 | Photographs showing the cuboid containing a spiral channel printed with the GelMA/HAMA (5.0%/3.0%) ink, followed by the digestion using Hase (1,000 U mL^{-1}) for 24 h. The color dye was perfused into the open channel to aid visualization.

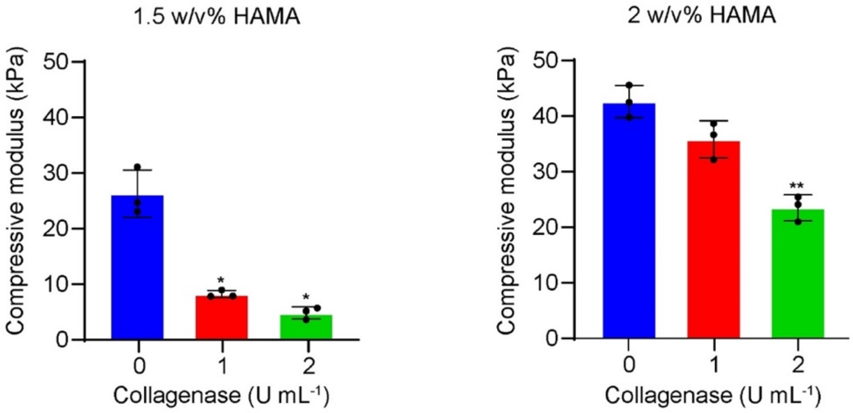

Supplementary Fig. 15 | Compressive moduli of hydrogel constructs made of HAMA/GelMA (2.5% of GelMA) inks at various HAMA concentrations (1.5% and 2.0%), digested with collagenase (0, 1, and 2 U mL^{-1}) for 24 h. $n = 3$; two-tailed student's t-test; *$p < 0.05$, **$p < 0.01$, (compared with the group without digestion). Data are presented as mean values ± SDs. Source data are provided as a Source Data file.

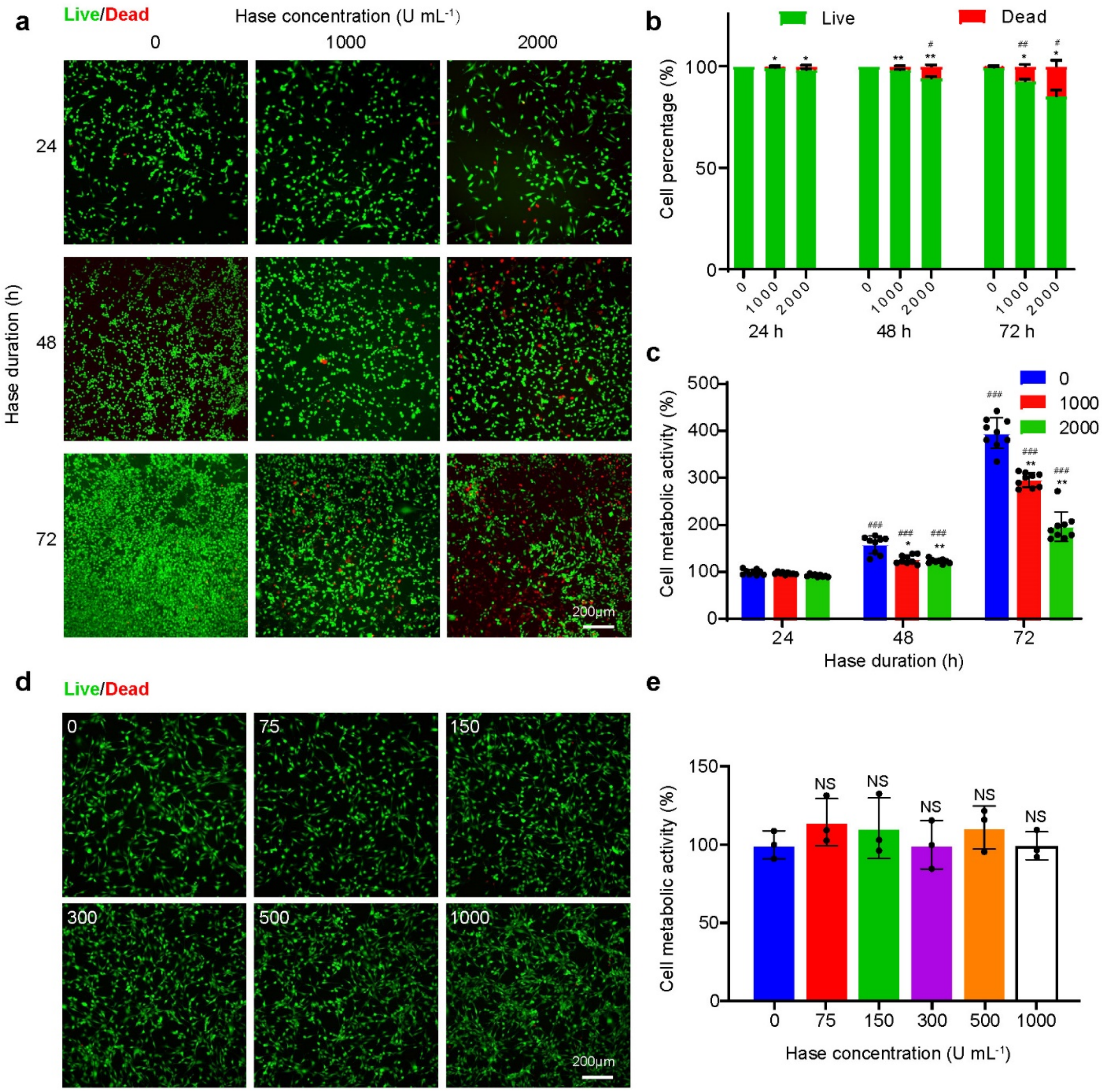

Supplementary Fig. 16 | Cytocompatibility analyses of Hase digestion on 2D-cultured NIH/3T3 fibroblasts. **a**, Micrographs showing live (green)/dead (red) staining of the cells treated for different durations (24, 48, and 72 h) with various concentrations of Hase (0, 1,000, and 2,000 U mL^{-1}). **b**, Corresponding quantitative analyses of the percentages of live/dead cells. **a** and **b**, $n = 3$; one-way ANOVA; $^{*}p < 0.05$, $^{**}p < 0.01$ (compared with the group without Hase treatment but with the same treatment duration); $^{\#}p < 0.05$, $^{\#\#}p < 0.01$ (compared with the corresponding results of the same Hase concentration and treated for 24 h). Data are presented as mean values ± SDs. **c**, Quantitative results of MTS assay of the cells treated for different durations (24, 48, and 72 h) and under various concentrations of Hase (0, 1,000, and 2,000 U mL^{-1}). $n = 9$; one-way ANOVA; $^{*}p$

< 0.05, $^{**}p < 0.01$ (compared with the group without Hase treatment but with the same treatment duration); $^{\#}p < 0.05$, $^{\#\#}p < 0.01$, $^{\#\#\#}p < 0.001$ (compared with the corresponding results of the same Hase concentration and treated for 24 h). Data are presented as mean values ± SDs. **d**, Micrographs showing live (green)/dead (red) staining of the cells treated with different concentrations of Hase (0, 75, 150, 300, 500, and 1,000 U mL^{-1}) for 24 h. Images are representatives of $n = 3$ independent experiments. **e**, Quantitative results of MTS assay of the cells treated with different concentrations of Hase (0, 75, 150, 300, 500, and 1,000 U mL^{-1}) for 24 h. $n = 3$; two-tailed student's t-test; NS, not significant (compared with the group without Hase treatment). Data are presented as mean values ± SDs. Source data are provided as a Source Data file.

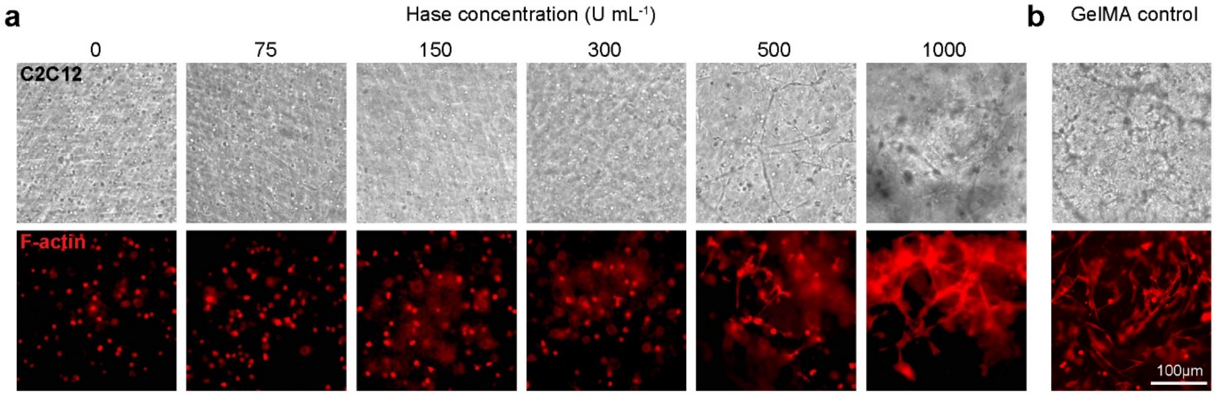

Supplementary Fig. 17 | Bright-field and fluorescence micrographs showing cell morphologies and F-actin staining (red) of C2C12 myoblasts (**a**) bioprinted with the GelMA/HAMA (7.5%/1.5%) bioink and treated by 0, 75, 150, 300, 500, and 1,000 U mL^{-1} of Hase for 24 h, or (**b**) cast using the pure GelMA (7.5%) bioink, followed by 3 days of culture after digestion or casting. Images are representatives of n = 3 independent experiments.

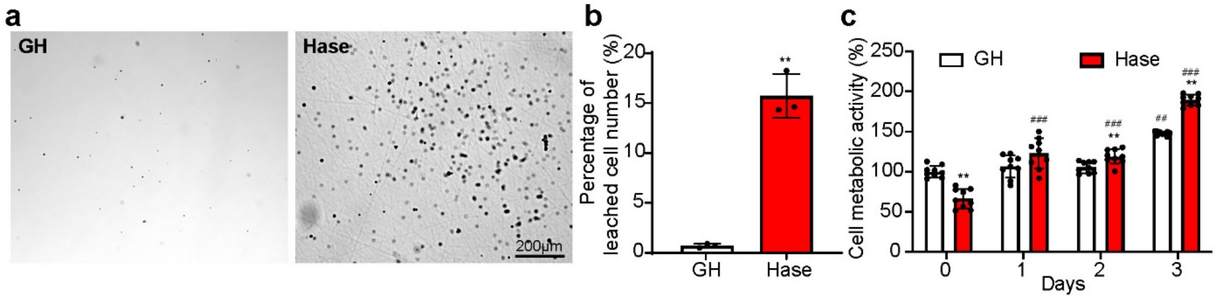

Supplementary Fig. 18 | Leaching study of NIH/3T3 fibroblasts from bioprinted GelMA/HAMA (7.5%/1.5%) samples. **a**, Bright-field micrographs of cells leached out from the bioprinted constructs and floated in the media without Hase treatment (GH group) or immediately after Hase digestion (1,000 U mL^{-1}, 24 h). Images are representatives of $n = 3$ independent experiments. **b**, Corresponding quantitative results of the percentages of leached cells. $n = 3$; two-tailed student's t-test; $^{**}p < 0.01$ (compared with the GH group. Data are presented as mean values ± SDs. **c**, Quantitative results of MTS assay of the cells within the bioprinted GelMA/HAMA (7.5%/1.5%) constructs without Hase treatment and digested with Hase (1,000 U mL^{-1}, 24 h) after 0, 1, 2, and 3 days. $n = 9$; one-way ANOVA; $^{**}p < 0.01$; one-way ANOVA (compared with the GH group but at the days after); $^{\#\#\#}p < 0.001$ (compared with the corresponding results of the day 0 and the same Hase treatment condition). Data are presented as mean values ± SDs. GH is composed of GelMA/HAMA (7.5%/1.5%), and Hase indicates the groups of GelMA/HAMA treated with Hase (1,000 U mL^{-1}) for 24 h. Source data are provided as a Source Data file.

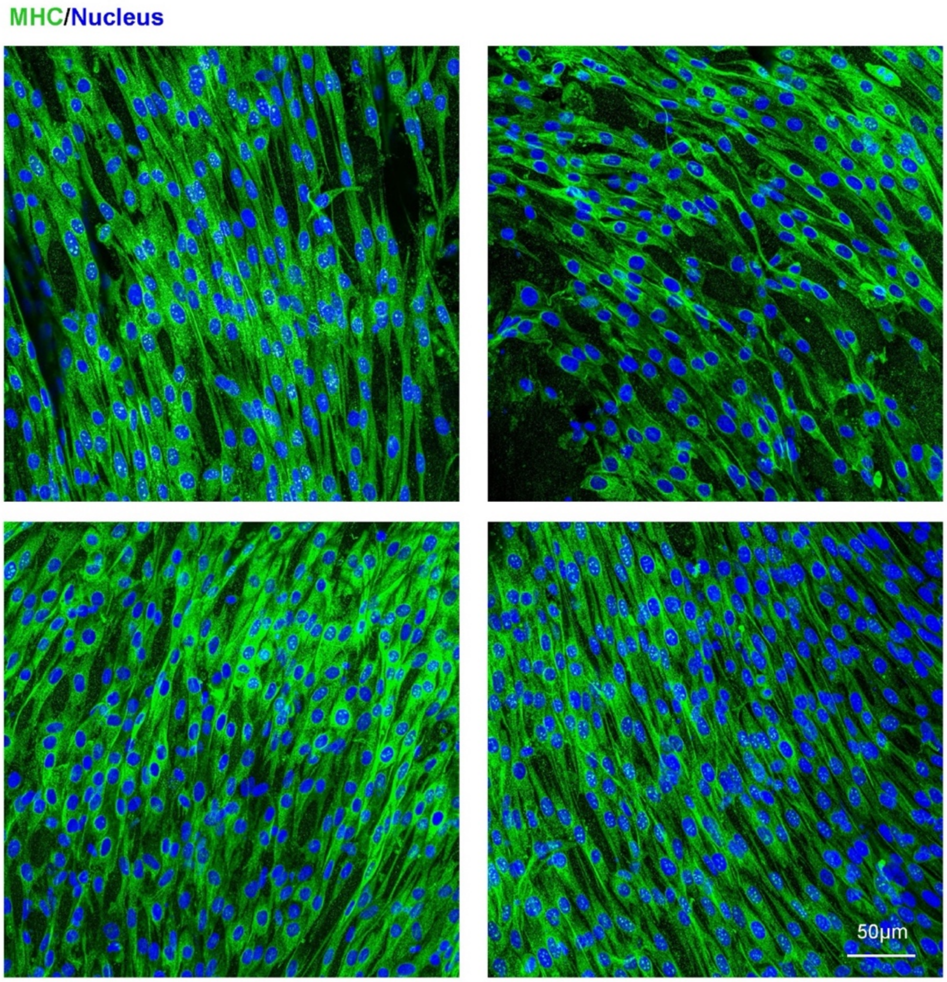

Supplementary Fig. 19 | Micrographs of MHC (green) staining of C2C12 cells cultured in the constructs bioprinted from GelMA/HAMA (7.5%/1.5%) with Hase digestion (1,000 U mL^{-1}, 24 h) at the day 14 after myogenic differentiation, presenting aligned myotubes in most if not all of the randomly captured images at different locations of multiple samples. Images are representatives of n = 3 independent experiments.

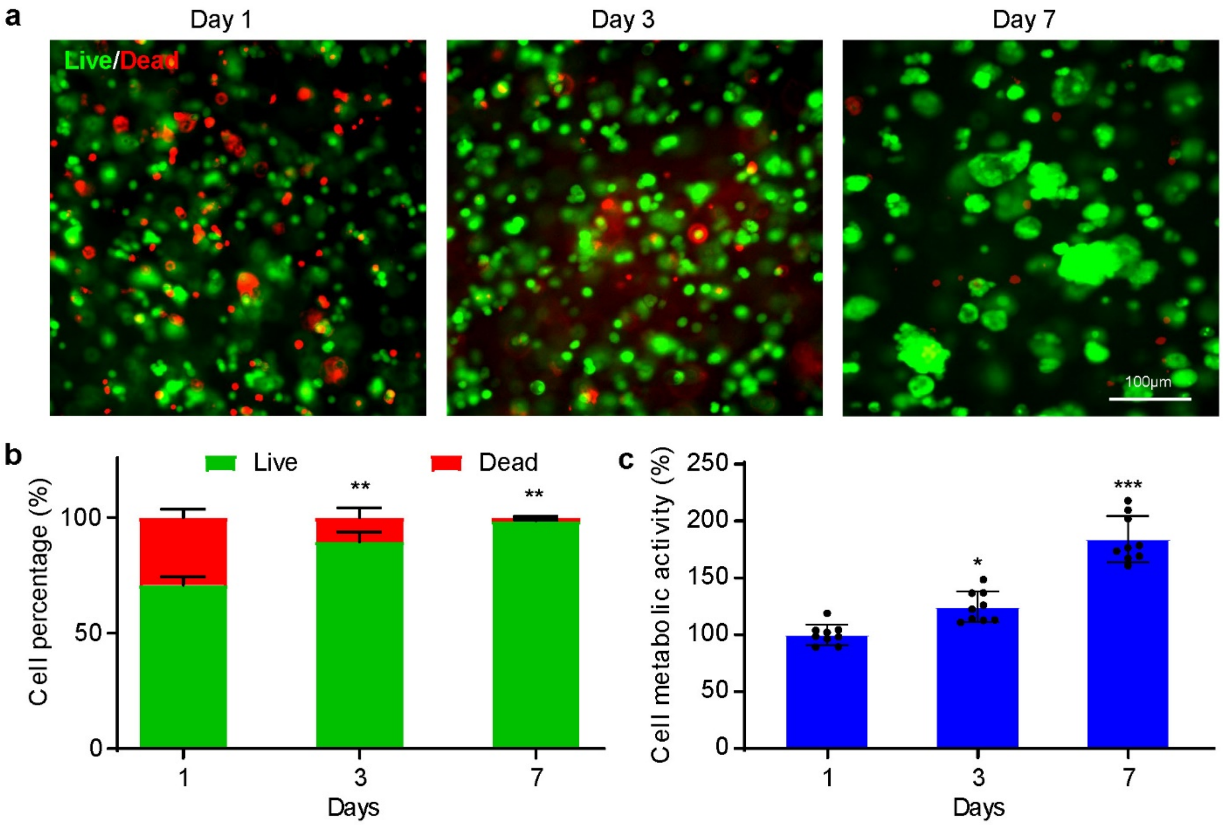

Supplementary Fig. 20 | HepG2/C3A cells cast in 5.0% GelMA. **a**, Micrographs showing live (green)/dead (red) staining of HepG2/C3A cells encapsulated in constructs cast with 5.0% GelMA at days 1, 3, and 7 of culture. Images are representatives of n = 3 independent experiments**.** **b**, Corresponding quantitative analyses of the percentages of live/dead cells. **c**, Quantitative results of MTS assay showing metabolic activities of the HepG2/C3A cells. $n = 3$ (**b**), $n = 9$ (**c**); two-tailed student's t-test; *$p < 0.05$, **$p < 0.01$, ***$p < 0.001$ (compared with the corresponding results of the day 1). Data are presented as mean values ± SDs. Source data are provided as a Source Data file.

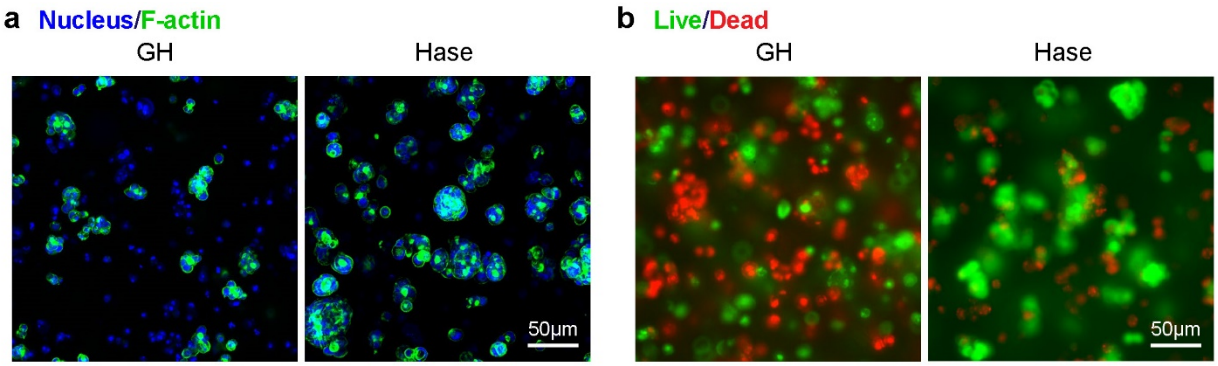

Supplementary Fig. 21 | Confocal immunofluorescence images showing **a,** F-actin staining (green) with nuclei counterstaining (blue), and **b**, live (green)/dead (red) staining, of HepG2/C3A cells cultured in bioprinted GelMA/HAMA (5.0%/1.5%) constructs without or with Hase digestion (1,000 U ml^{-1}, 24 h) at day 14 after digestion. Images are representatives of $n = 3$ independent experiments. GH is composed of GelMA/HAMA (5.0%/1.5%), and Hase is the group of GelMA/HAMA treated with Hase (1,000 U mL^{-1}) for 24 h.

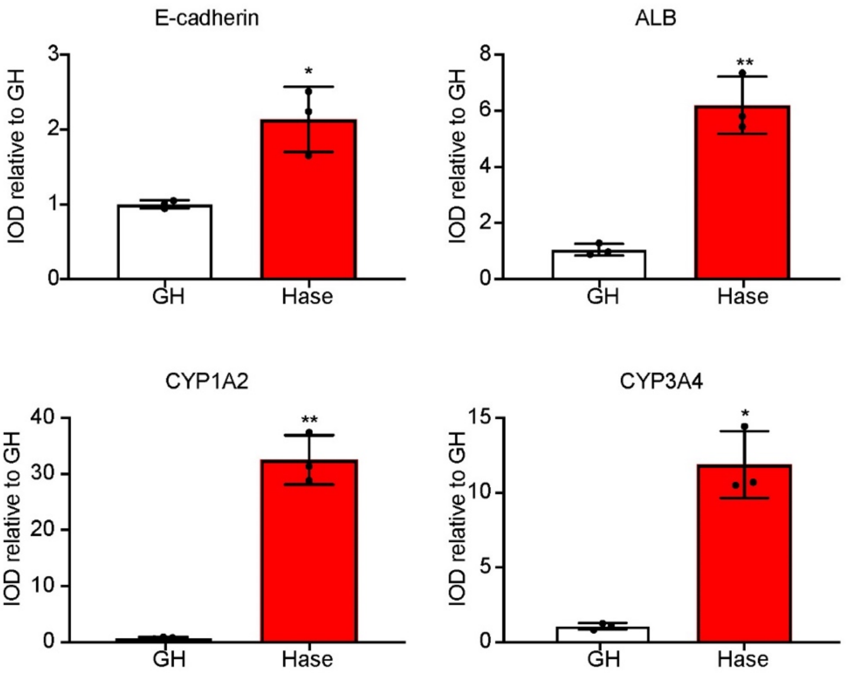

Supplementary Fig. 22 | Quantitative results of IODs of E-cadherin, ALB, CYP1A2, and CYP3A4 of HepG2/C3A cells encapsulated in constructs bioprinted with GelMA/HAMA (5.0%/1.5%) without or with 24 h of Hase digestion (1,000 U ml^{-1}, 24 h) at day 14 after digestion. All IODs are relative to the corresponding results of the GH group. $n = 3$; two-tailed student's t-test; $*p < 0.05$, $**p < 0.01$ (compared with GH group). Data are presented as mean values ± SDs. GH is composed of GelMA/HAMA (5.0%/1.5%), and Hase is the group of GelMA/HAMA treated with Hase (1,000 U mL^{-1}) for 24 h. Source data are provided as a Source Data file.

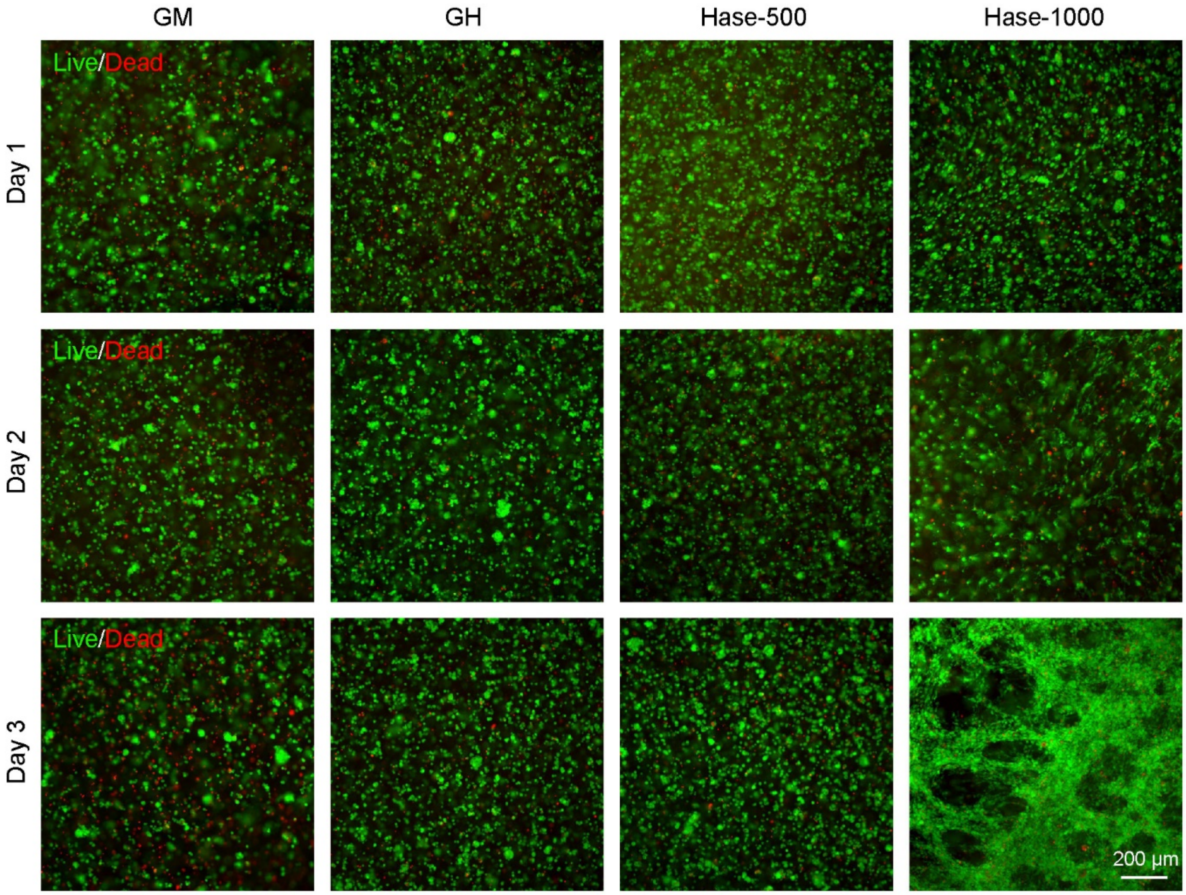

Supplementary Fig. 23 | Fluorescence micrographs presenting live (green)/dead (red) staining results of SNaPs cultured in 10% GelMA and GelMA/HAMA (2.5%/1.5%) constructs without or with Hase digestion (500 or 1,000 U mL^{-1}) for 24 h, at days 1, 2, and 3 after digestion. Images are representatives of $n = 3$ independent experiments**.** GM indicates the hydrogel made of 10% GelMA. GH is composed of GelMA/HAMA (2.5%/1.5% for brain-like tissue), and Hase-500 and Hase-1000 indicate the groups of GelMA/HAMA treated with Hase (500, 1,000 U mL^{-1}, respectively) for 24 h.

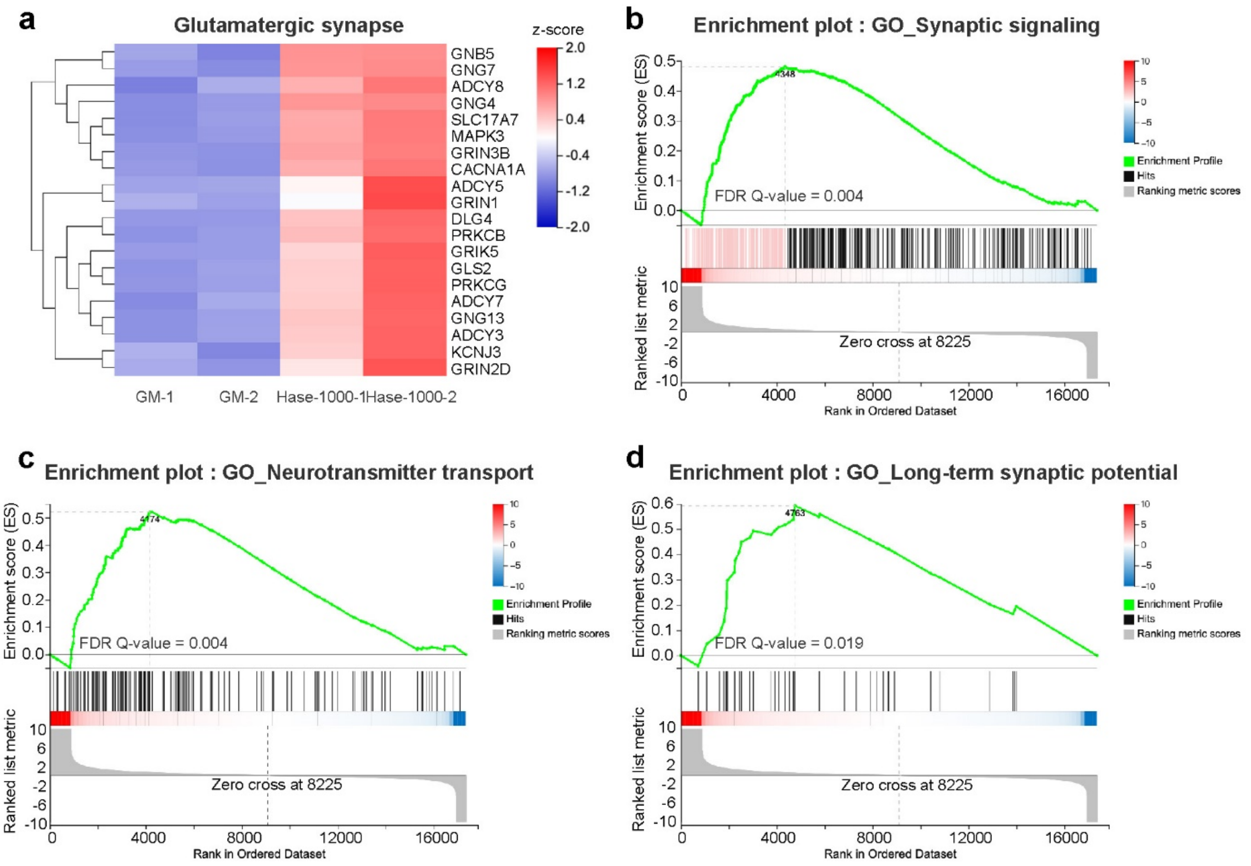

Supplementary Fig. 24 | Transcriptomic assessments of SNaPs cultured in bioprinted 10% GelMA and GelMA/HAMA (2.5%/1.5%) with 1,000 U mL^{-1} of Hase digestion for 24 h after 4 weeks of differentiation. **a**, Heatmap showing differential genes expressions relating to glutamatergic synapse. **b-d**, GSEA analyses of (**b**) synaptic signaling, (**c**) neurotransmitter transport, and (**d**) Long-term synaptic potential.

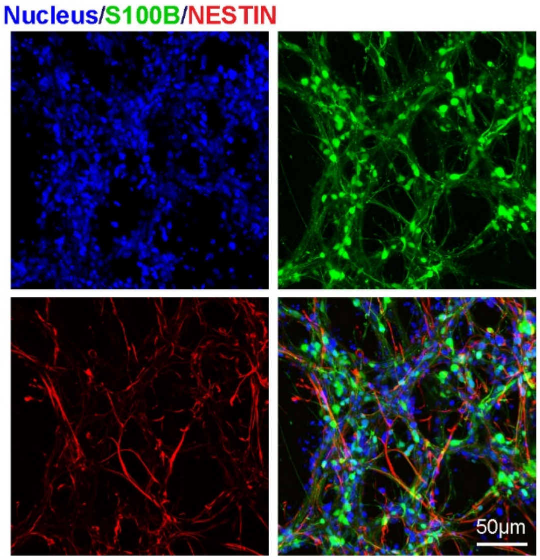

Supplementary Fig. 25 | Confocal immunofluorescence images displaying S100B (green), NESTIN (red), and nuclei (blue) staining of bioprinted SNaPs after 4 weeks of differentiation in GelMA/HAMA (2.5%/1.5%) constructs digested by Hase (1,000 U mL^{-1}) for 24 h post-bioprinting. Images are representatives of n = 3 independent experiments.

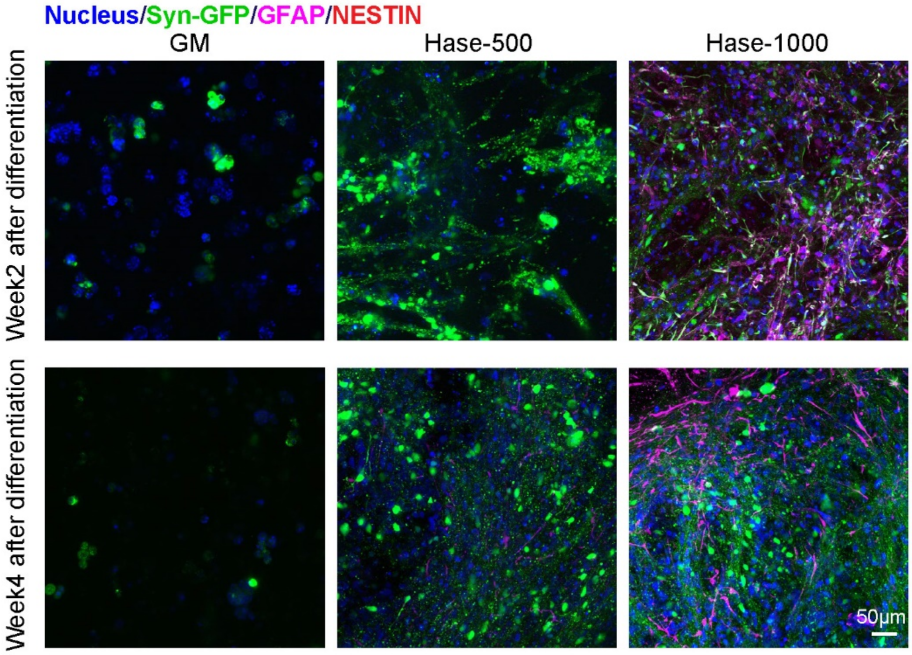

Supplementary Fig. 26 | Immunostaining results of SNaPs (green), GFAP (magenda), NESTIN (red), and nuclei (blue) of bioprinted SNaPs after 2 and 4 weeks of differentiation in 10% GelMA, GelMA/HAMA (2.5%/1.5%) with 500 U mL^{-1} or 1,000 U mL^{-1} of Hase digestion for 24 h post-bioprinting. Images are representatives of n = 3 independent experiments. GM indicates the hydrogel made of 10% GelMA. Hase-500 and Hase-1000 indicate the groups of GelMA/HAMA (2.5%/1.5%) treated with Hase (500, 1,000 U mL^{-1}, respectively) for 24 h.

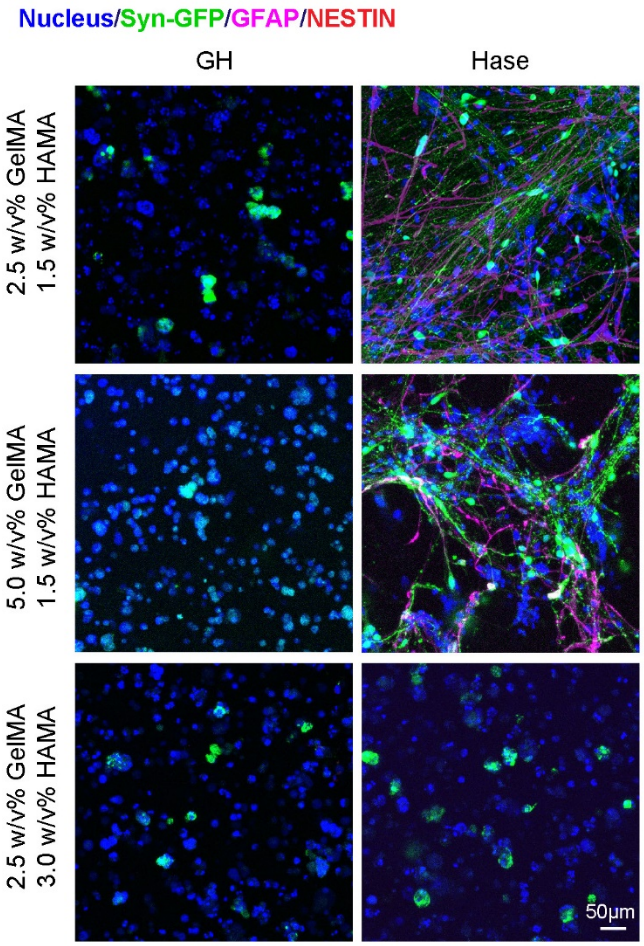

Supplementary Fig. 27 | Immunostaining results of SNaPs (green), GFAP (magenda), NESTIN (red), and nuclei (blue) of bioprinted SNaPs after 4 weeks of differentiation in constructs made of GelMA/HAMA (2.5%/1.5%) with or without Hase (1,000 U mL^{-1}) digestion for 24 h post-bioprinting. Images are representatives of $n = 3$ independent experiments.

Supplementary Table 1 | Two-way ANOVA of the quadratic response surface model.

Source	*Sum of Squares*	*df*	*Mean Square*	*F-value*	*P-value*
Model	4.69E+05	11	42587.79	463.23	< 0.0001
A-GelMA	75272.64	1	75272.64	818.74	< 0.0001
B-Digestion Time	85329.98	1	85329.98	928.13	< 0.0001
C-HAase	71072	1	71072	773.05	< 0.0001
D-HAMA	1.88E+05	1	1.88E+05	2044.48	< 0.0001
AB	6929.6	1	6929.6	75.37	< 0.0001
AC	8354.8	1	8354.8	90.87	< 0.0001
AD	26294	1	26294	286	< 0.0001
BD	32113.34	1	32113.34	349.3	< 0.0001
CD	24243.24	1	24243.24	263.69	< 0.0001
B^2	11263.5	1	11263.5	122.51	< 0.0001
C^2	12157.87	1	12157.87	132.24	< 0.0001
Residual	20042.32	218	91.94		
Lack of Fit	15816.03	60	263.6	9.85	< 0.0001
Pure Error	4226.29	158	26.75		
Cor Total	4.89E+05	229			
Std. Dev.	9.59				
Mean	48.63				
C.V. %	19.72				
R^2	0.959				
Adjusted R^2	0.9569				
Predicted R^2	0.9544				
Adeq Precision	78.2841				

Note: A=GelMA concentration (%); B=Digestion time (h); C=Hase concentration (U mL^{-1}); D=HAMA concentration (%).

Supplementary Table 2 | Experimental confirmation runs for validating the mathematical model.

GelMA (w/v%)	Digestion time (h)	Hase (U mL^{-1})	HAMA (w/v%)	Predicted Modulus Mean (kPa)	95% PI low (kPa)	Actual Modulus Mean (kPa)	95% PI high (kPa)
2.62	4.2	966	1.54	6.13	0.00	7.88	17.72
2.64	9	970	2.4	14.65	3.16	17.53	26.15
2.50	23.48	2.66	1.86	31.33	19.41	25.13	43.26
6.82	8.95	719.46	2.50	65.80	53.86	72.10	77.75
5.45	2.23	918.89	2.97	107.79	98.43	113.63	121.16

Supplementary Table 3 | BET surface areas and average pore sizes of control and Hase-digested samples.

Sample	Specific surface area (m^2 g^{-1})	Average pore size (nm)
Control	204	54
Hase	295	38

Supplementary Table 4 | Primers used for the RT-PCR of HepG2/C3A functionality evaluations.

Gene	Forward primer (5'-3')	Reverse primer (5'-3')
MKI67	TTACAAGACTCGGTCCCTGAA	TTGCTGTTCTGCCTCAGTCTT
ALB	GATGAGATGCCTGCTGACTTGC	CACGACAGAGTAATCAGGATGCC
AFP	GCAGCCAAAGTGAAGAGGGAAGA	GTCATAGCGAGCAGCCCAAAG AAG
CASP8	CTTGGATGCAGGGGCTTTGACC	GTTCACTTCAGTCAGGATGG
